# Automated monitoring of behaviour in zebrafish after invasive procedures

**DOI:** 10.1038/s41598-019-45464-w

**Published:** 2019-06-21

**Authors:** Anthony G. Deakin, Jonathan Buckley, Hamzah S. AlZu’bi, Andrew R. Cossins, Joseph W. Spencer, Waleed Al’Nuaimy, Iain S. Young, Jack S. Thomson, Lynne U. Sneddon

**Affiliations:** 1 Electrical Engineering and Electronics, University of Liverpool, Liverpool, L69 3BX UK; 2 Department of Evolution, Ecology and Behaviour, Institute of Integrative Biology, The BioScience Building, University of Liverpool, Liverpool, L69 7ZB UK; 3 Department of Functional and Comparative Genomics, Institute of Integrative Biology, The BioScience Building, University of Liverpool, Liverpool, L69 7ZB UK; 4 School of Environmental Sciences, University of Liverpool, Nicholson Building, Brownlow Street, Liverpool, L69 3GP UK

**Keywords:** Behavioural methods, Data processing

## Abstract

Fish are used in a variety of experimental contexts often in high numbers. To maintain their welfare and ensure valid results during invasive procedures it is vital that we can detect subtle changes in behaviour that may allow us to intervene to provide pain-relief. Therefore, an automated method, the Fish Behaviour Index (FBI), was devised and used for testing the impact of laboratory procedures and efficacy of analgesic drugs in the model species, the zebrafish. Cameras with tracking software were used to visually track and quantify female zebrafish behaviour in real time after a number of laboratory procedures including fin clipping, PIT tagging, and nociceptor excitation via injection of acetic acid subcutaneously. The FBI was derived from activity and distance swum measured before and after these procedures compared with control and sham groups. Further, the efficacy of a range of drugs with analgesic properties to identify efficacy of these agents was explored. Lidocaine (5 mg/L), flunixin (8 mg/L) and morphine (48 mg/L) prevented the associated reduction in activity and distance swum after fin clipping. From an ethical perspective, the FBI represents a significant refinement in the use of zebrafish and could be adopted across a wide range of biological disciplines.

## Introduction

To ensure the validity and reliability of scientific studies, it is vital to use normal individuals in experiments but also to maintain good health during and after laboratory procedures. Experimental guidelines and legislation of many countries state that animals should be held in optimal conditions for that species with the absence of adverse events causing pain and/or stress. Invasive experimental procedures could cause stress and/or pain without those factors being intrinsically important to the objectives of the study and if normal behaviour or physiology is affected then the response to these events may confound data collection^1,2^. In this case the researcher may wish to minimize these confounding factors but needs to have the capacity to recognize these phenomena in order to do so. Ethical guidelines also require that welfare is monitored during experiments so that animals are kept in optimal wellbeing where possible. Depending on the species being studied, however, monitoring for normal behaviour during experiments can present distinct challenges.

Zebrafish (*Danio rerio*) are a significant model organism in biological and medical research^3^ with fish second only to mice in numbers used in regulated procedures in the UK (https://www.gov.uk/government/statistics/statistics-of-scientific-procedures-on-living-animals-great-britain-2017). Their use is increasing (e.g. 28% increased use of fish species in experiments to ~1.3 million 2008 to 2011 (http://eur-lex.europa.eu/legal-content/EN/TXT/?uri=CELEX:52013SC0497). Whilst it used to be considered that fish did not experience pain, recent evidence supports the consensus that fish, including zebrafish, have the potential to experience pain or nociception and stress^4–6^. Therefore concern for fish welfare in experimentation stems from both scientific and ethical requirements. The principles of Replacement, Reduction and Refinement (3Rs) were developed over 50 years ago as a framework for humane animal research and are embedded in national and international legislation regulating the use of animals in scientific procedures (www.nc3rs.org.uk/the-3Rs). Therefore it is necessary during animal experiments to minimize factors that may challenge welfare and to intervene when necessary (e.g. providing appropriate pain relief). However, the scientific community employing fish models currently have no reliable, convenient and low cost means of automatically assessing when a fish subject is experiencing pain and/or stress. Although some researchers have provided very useful guides and terminology on characterizing zebrafish welfare by eye, these tend to focus on overt signs of ill health (e.g. lesions, lack of movement, scoliosis)^7,8^. However, many prey animals may only exhibit subtle behavioural changes^9–11^ when first experiencing negative welfare that are not discernible using the human eye thus an automated monitor that can track behavioural changes may allow us to intervene sooner to remedy any welfare challenges. However, there may also be overt changes in behavior that can allow assessment of the intensity of change from normal. Several studies demonstrate normal, healthy zebrafish are usually constantly swimming, using mid water and all areas of their tank as opposed to behavior after potentially painful treatment where zebrafish increase their use of the bottom of the tank, reduce their swimming and activity^6,12–16^.

A system capable of automatically measuring pain from behavioural responses without human error or bias would be invaluable in helping to assure experimental fish behaviour was normal and could gauge possible pain after invasive procedures. Additionally, given the high numbers of fish employed in such studies, this could reduce staff time on making such assessments (e.g. large-scale zebrafish facilities with hundreds of tanks), which are required on a daily basis, and thereby enhance researcher productivity. In order to prototype and evaluate such an automated system, a range of experiments were designed to present normal healthy, acclimated female zebrafish with a number of invasive and stressful treatments that could be used to assess their behavioural responses compared with undisturbed controls and sham treated (anaesthetised and handled) individuals. Whilst individual fish may behave differently when they are in a group or shoal than when they are held individually^12,13^, it is necessary to first investigate whether the effects of experimental treatments can be detected in a single fish before considering either a single treated fish within a group or a group of treated fish. Additionally, zebrafish are held individually after invasive procedures to allow recovery and healing (e.g. cardiac surgery^17^, optic nerve crush^18^ and spinal lesions^19^) and are not provided with pain relief. Therefore, it is especially important to assess normal behaviour and alterations in individually held fish subject to a range of invasive procedures. This allows the testing of a range of drugs with analgesic properties to determine which prevents the behavioural changes after fin clipping, a procedure performed routinely for genomic screening in zebrafish^14^. This allowed us to develop the Fish Behaviour Index (FBI) based upon the behavioural changes and further test whether the FBI scores were correlated with other treatments and the assessment of a trained person.

## Methods

### Subjects and husbandry

**Figure 1:**
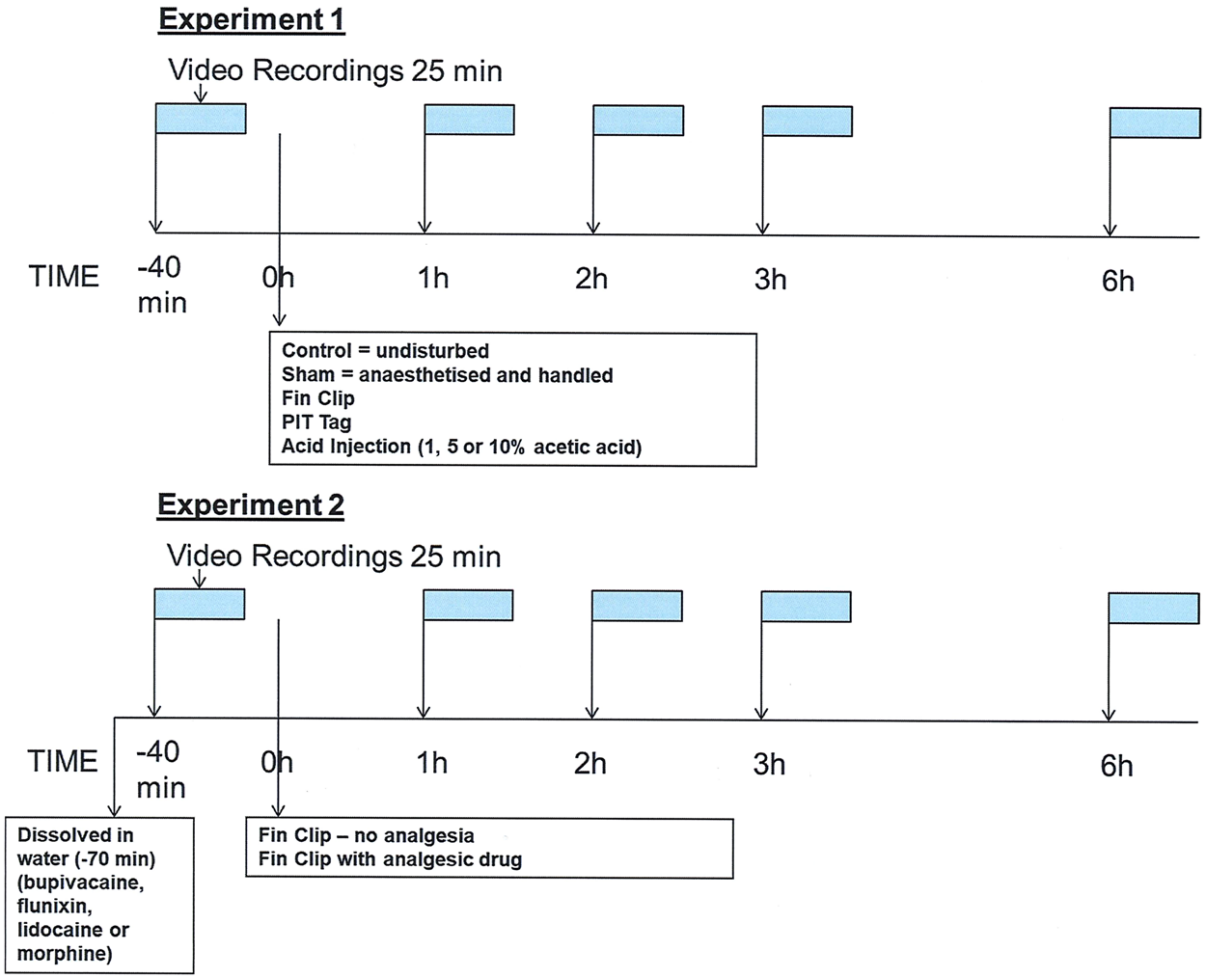
A schematic representation of Experiment 1 and Experiment 2. Video recordings were made at each time point: pretreatment (−40 min), followed by anaesthesia and treatment (fin clip, PIT tag or subcutaneous injection of acetic acid) at 1, 2, 3 and 6 h after treatment. Control zebrafish were undisturbed and sham treated individuals were anaesthestised and handled. In experiment 2, one analgesic drug at one dose was dissolved in the tank water (bupivacaine at 0.25, 0.5 and 1 mg/L; flunixin at 2, 4 and 8 mg/L; lidocaine at 5 mg/L and morphine at 3 and 48 mg/L) 30 min prior to the first pre-treatment recording.

Eight month old female zebrafish (*D. rerio*) of AB strain (n = 126; mean size 0.92 g ± 0.08) were randomly selected from the University of Liverpool aquarium in-house breeding project for the experiments. Females were used to prevent any confounding effects of gender on behavior and physiology as has been found in studies conducted in our laboratory^20^ and elsewhere^21–25^. Stock fish were maintained in a semi-closed recirculation system in 10 L tanks (Aquatic Habitats, Florida USA) at 27 ± 1 °C, with constant aeration on a 14:10 h light:dark cycle. Fish were selected at random, netted carefully into a 3 L tank and transferred from stock tanks to a semi-closed recirculation system consisting of two parallel rows of glass tanks (20 × 30 × 20 cm; n = 1 fish per tank). Each tank was fitted with an identical, external laminated printout of a green plant background; this allowed the easy detection of the focal fish by an in house tracking system^26^ due to the enhanced contrast provided by the green background. All tanks were supplied with filtered water (NH_3_ = < 0.01 mg/L, NO_2_ = < 0.01 mg/L, NO_3_ = < 5 mg/L) maintained at a temperature of 27 ± 1 °C, under a 14:10 light:dark regime with aeration provided by an aerated 200 L biological filter with one third of the water replaced weekly. Fish were acclimatized in their individual experimental tank for two weeks prior to experimentation and fed twice daily *ad libitum* with a commercial tropical ornamental flake (TetraMin, Tetra, Melle, Germany). All fish used in experiments had fed consistently when food was presented for at least seven days prior to experimentation commencing. Fish were in chemical (through shared water) and visual contact with adjacent tanks so had social contact until the evening prior to experimentation when two opaque pieces of plastic were placed in between tanks to visually isolate the test individuals and the inflow was turned off 30 min prior to the commencement of experiments. All methods were performed in accordance with the relevant guidelines and regulations. A schematic representation of the procedures and timings used in two experiments is shown on Fig. 1.

### Experiment 1: The impact of common procedures on the behaviour of zebrafish

The effect of laboratory procedures on the behaviour of female zebrafish was tested against control (undisturbed) and sham handled groups (anaesthetized and handled in a similar manner and time frame but no invasive treatment). We did not perform any sham injections of non-painful saline since previous studies demonstrate there are no differences between control and sham injected fish^27–30^ (these studies demonstrate the injection itself does not significantly affect subsequent behavior). Fish were randomly assigned to one of seven treatment groups (n = 7 for each group; n = 49 total): Control; Sham; five noxiously-stimulated groups (1–3) injected subcutaneously with either 1%. 5% or 10% acetic acid into the lips (concentrations used in previous studies)^15,29^; 4. PIT tag insertion through the abdomen; and 5. Fin clip where 40% of the caudal fin was removed using sterile surgical scissors as described in The Zebrafish Handbook (http://zfin.org/zf_info/zfbook/chapt7/7.8.html). Control fish were left undisturbed for the duration of the experiment whereas after the first behavioral recording all treatment fish were carefully netted and transferred to a 1 L beaker containing 500 ml of aerated water dosed with benzocaine (0.033 g l-1; Sigma-Aldrich Co., UK) where fish were anaesthetized. Benzocaine was used as it has short lasting analgesic properties and fish were taken to deep plane anesthesia where they were unconscious during the noxious treatments shown by a lack of reflex responses^31^. After anesthesia the sham treatment group were handled (netted, placed in wet paper towels and returned to their home tank) for the same duration as the other procedures but no invasive procedure applied. During anesthesia the acetic acid groups were injected with the appropriate concentration of acetic acid using a sterile gastight syringe and needle (34 g; Hamilton; Bonaduz, Switzerland). Acid was injected subcutaneously into the frontal lips using 2 ɥl per lip. PIT tag treatment fish were orientated upside down and a sterile 20 gauge needle used to make an incision in the abdominal cavity so that a 4 mm PIT tag (Loligo systems; Denmark) could be careful inserted. All fish were returned to their home tank after the procedure and allowed to recover from the anesthesia for 1 h before observations commenced.

Experiments were conducted at the same time each day (10 am) to prevent any diurnal effects. Behaviour was recorded for 25 minute periods at the time points 40 minutes pre procedure, and then 1, 2, 3 and 6 hours after the procedure. These time points were chosen as fish subject to noxious stimulation usually show an initial adverse response up until 3 hours then recovery by 6 hours in acetic acid tests^15,32^. Two industrial IDS USB 3.0 colour video cameras (IDS; Obersulm, Germany) fitted with a 25 mm monofocal lens and connected to a computer (HP compact elite 8300; Palo Alto, Ca) running the fish tracking software^26^ were used to accurately track the movements of individual zebrafish. Cameras positioned above and to the front of the focal tank were used to track the 3D trajectories of fish. Cameras positioned dorsally were mounted on a sliding gantry 1.4 m above the two parallel rows of 9 tanks; this enabled the cameras to be moved from tank to tank without disturbing the fish. Cameras positioned to the side were attached to tripods 1.4 m away from the focal tanks and were moved manually between tanks. One tank on either side of the rows was randomly assigned to a treatment group with cameras moved the previous evening which allowed two fish per day to be recorded; as treatments were randomized this prevented any confounding effects of order in the experiment. Data files generated by the 3D tracking software were then processed with software in MATLAB^26^. This software allowed the extraction of 20 characteristics (Table 1) from an individual’s 3D coordinates. Videos were assessed blind until data had been collected then the identity of the fish was related to the data set.

**Table 1:** List of zebrafish behavioural characteristics extracted from the tracking and behavioural analysis software (those with abbreviated labels on Supplementary Fig. S8).

Distance (cm)	Speed (cm/sec^−1^)
Total distance travelled (dist) Total distance travelled top half of tank Total distance travelled bottom half of tank (dis bot)	Average speed (speed) Average speed top half of tank Average speed bottom half of tank (av speed bot) Max speed top half of tank (max speed top) Max speed bottom half of tank
**Manoeuvring**	**Acceleration (cm/sec** ^**−2**^ **)**
% time spent active (active) Average number of turns (turns) % of movements exceeding 12 cm/sec^−1^ at angles above 45 degrees	Average acceleration (accel) Average deceleration (decel) Average acceleration top half of tank Average acceleration bottom half of tank (ava cc bot) Max acceleration top half of tank (max acc top) Max acceleration bottom half of tank
**Space use**	
% time spent in bottom half of tank (timebot) % tank explored (occupy) % time spent near walls	

### Experiment 2: The prevention of pain related behaviour via immersion analgesics

Out of the five invasive procedures, the fin clip resulted in some of the most consistent changes in behaviour without recovery at 6 h. Therefore, the fin clip was chosen as the procedure by which the efficacy of immersion analgesics would be tested. Three categories of analgesic including nonsteroidal anti-inflammatory drugs (NSAIDs), local anesthetics and opioids were tested at a range of doses and extrapolated from studies using fish or mammals^14,15,27–29,31,32^. The doses applied were: NSAID flunixin 2, 4, and 8 mg/L; opioid morphine 3 and 48 mg/L; local anaesthetics bupivacaine 0.25, 0.5 and 1 mg/L and lidocaine 5 mg/L.

Zebrafish were randomly assigned to each treatment group. At the start of the experiment (09:30) the water flow to the experimental tanks was switched off and the analgesic administered evenly throughout the tank. All analgesics were administered prior to the fin clip so that the effect of the drug on normal behaviour could be determined in the pre-treatment recording. Once the analgesic had been administered the same data collection methods as outlined in experiment 1 were carried out so that a behavioural profile could be obtained for the time points pre-treatment and 1, 2, 3 and 6 h afterwards.

### Statistical analyses

Data were analysed using SPSS software (Version 24). A principal component analysis (PCA) was used to explore which characteristics were responsible for the differences between control fish and those experiencing a potentially painful intervention; the PCA was based on 20 characteristics extracted from the xyz coordinates of individuals. The control values consisted of all control values and all pre-treatment values from the other experimental groups. All ‘pain’ characteristics were taken from the 2, 3 and 6 hour time points from fin clip, PIT tag, and acid lip groups. PCA data had a Kaiser-Meyer-Olkin value of 0.781 and a Bartlett’s test of sphericity value of <0.0001 indicating the appropriate use of PCA to examine correlations. Data was rotated using direct Oblimin with Kaiser normalization. Once the three most important characteristics had been selected the data was then assessed for homogeneity of variance and normality. After transformation Experiment 1 data was normally distributed as assessed by Shapiro-Wilk’s test and displayed homogeneity of variance as assessed by Levene’s test. Data did not require transformation for experiment 2.

A mixed model ANOVA was used to investigate whether time, treatment, or their interaction had an influence on each of the behaviours in experiment 1 using repeated measures for individuals (results in Supplementary Table 2). Assumptions of sphericity were checked using Mauchly’s tests for the data for percentage time at bottom; none of the data sets exhibited sphericity and thus Greenhouse-Geisser (average speed, percentage tank explored) or Huynh-Feldt (percentage time spent at bottom of tank) corrections were applied based on estimated values of ε. Interactions were significant for all behaviours; for post-hoc analysis, therefore, simple effects of Time were determined, and separate one-way ANOVA tests conducted to examine simple effects of Treatment. In experiment 2 the fin clipped analgesic groups were compared with controls and with fin clip without analgesia since this allowed us to gauge the efficacy of the drug. If the drug was significantly different from control it was deemed not effective in preventing responses to the fin clip. Conversely, if the drug treated animals displayed behaviours that were similar to controls then the drug had been successful in preventing responses to fin clip. As described above, data sets failed the assumption of sphericity and therefore Greenhouse-Geisser (percentage time spent at bottom of tank) and Huynh-Feldt (average speed, percentage tank explored) corrections were utilised. The interaction of Treatment × Time was significant for average speed, and post-hoc analysis conducted as for experiment 1 (results in Table S3). However, for percentage time spent at bottom of tank and percentage tank explored, only the main effects of Time and Treatment were significant (Table S3). Post-hoc analyses were therefore conducted for each individual main effect separately, with Tukey (Treatment) and Bonferroni (Time) corrections as appropriate.

### Creation of FBI

**Figure 2:**
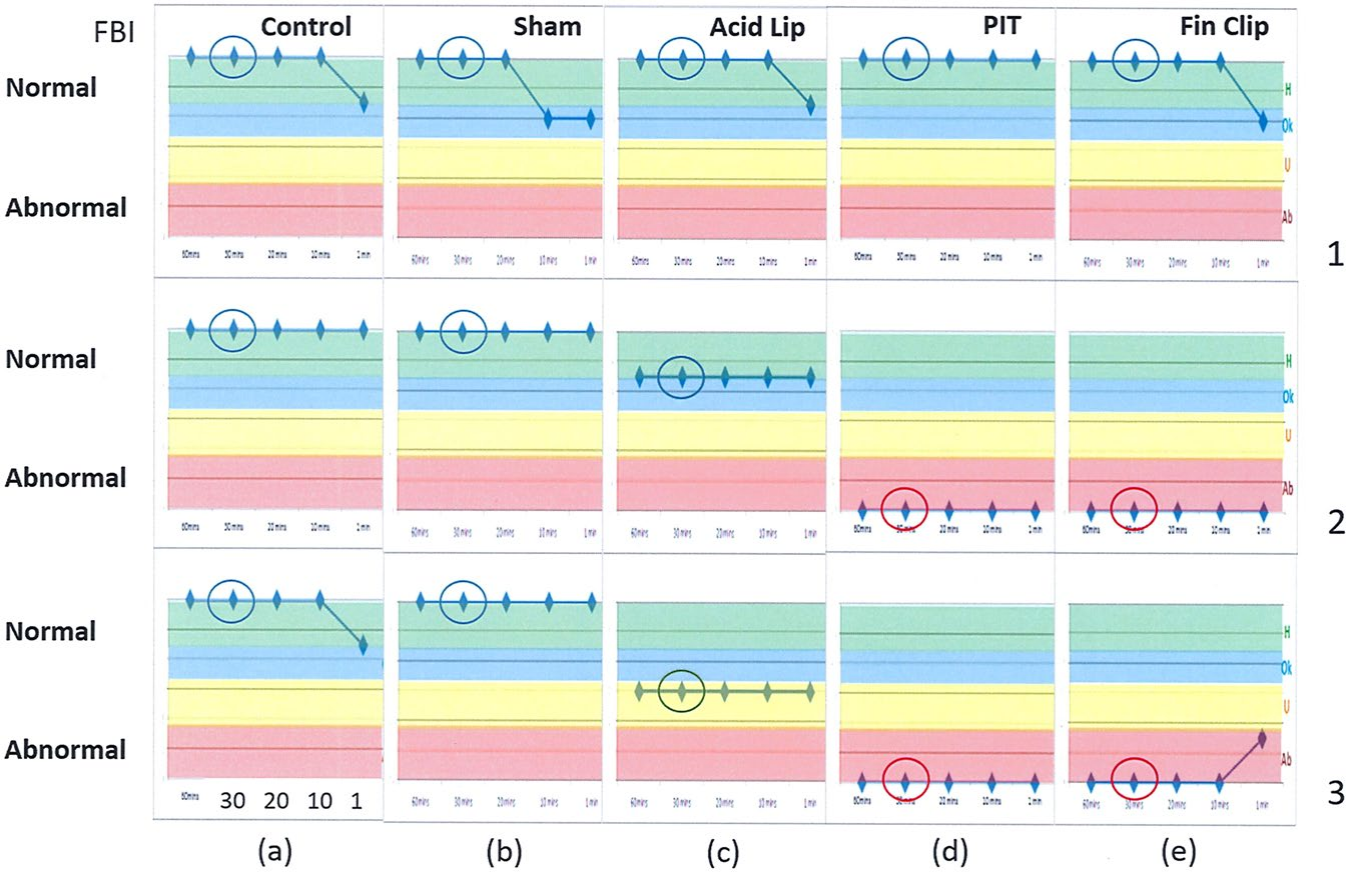
Examples of the Fish Behaviour Index (FBI; ranging 0 to 6) output before (1) and after (2, 3 h) treatment for 5 individual fish from 5 groups: (**a**) Control (**b**) Sham (**c**) Acid Lip (**d**) PIT (**e**) Fin Clip treated zebrafish over 3 hours in total (represented by 1, 2 and 3 at the end of each row), using 4 timescales each, from the latest 30 mins (circled) to the latest 20 mins, 10 mins and 1 min. Gradation of score (FBI) from green (Normal/Healthy), blue (Ok/Healthy), yellow (Unhealthy) to pink (Abnormal) provides a means of assessing how the subject’s behaviour differs from normal. Blue circles highlight Normal to Ok ratings whereas green circles refer to Unhealthy ratings and red circles highlight Abnormal ratings.

The FBI was designed to generate a scale in four broad categories, starting at normal and decreasing to an abnormal change in behaviour using generic labels for ease of reference: ‘Healthy’, ‘Ok’, ‘Unhealthy’ and ‘Abnormal’. We suggest these terms are easily understood and clear with Healthy and Ok not requiring concern, however, Unhealthy and Abnormal would require attention from carers and researchers. The timescales used to process acquired data from the videos were the latest 30, 20, 10 and 1 minutes, all updated once a minute in real time (SI Videos 1 and 2). The basic monitoring unit of 1 minute was thereby grouped into longer behavioural periods of 10, 20 and 30 minutes giving a range of timescales from very short (1 m) to relatively long (30 m). FBI is a combination of evaluations of “Activity” and of “Distance” travelled over these timescales providing a score which allows the behaviour of the fish to be scaled (Table 2). Activity was directly related to the parameter % tank explored and indirectly to % time spent in bottom of the tank and was calculated as the space utilization percentage (number of zones of the tank (1 to 9, out of 9) visited per minute with the horizontal and vertical axes of the tank each divided into three equal sections. Distance is the cumulative distance travelled in the timescale and relates directly to average speed. Not only are these significantly altered in the present study, these behaviours are known to be of scientific relevance (e.g.^12–14,33–39^ www.noldus.com/animal-behaviour-research/solutions/research-fish/stress-and-anxiety). Supplementary Fig. 1 illustrates FBI for a normal (untreated) fish monitored over 40 minutes separately from the 5 group experiments, giving latest 40, 30, 20, 10, 1 minute behaviour status (Supplementary Fig. 1(a)), plus a more detailed, deeper historical view of FBI over the 40 minutes with continuous 10 minute and 1 minute indicators (Supplementary Fig. S1(b)). Supplementary Fig S2 illustrates the data from which FBI is derived. Thus, the FBI is a live monitoring system to give a rating updated every minute and designed to characterize a subject’s behaviour as a combination of quantitative information and qualitative categories. As fish behaviour may vary considerably over different timescales, the FBI was designed to indicate fish responses to the stimuli over overlapping but independently evaluated timescales – short (1 min), medium (10 min) and long (30 min) - to provide a more complete picture of how behaviour varied. A medium timescale was considered significant for alerting to compromised welfare deviations requiring intervention (with e.g. analgesics, veterinary advice or the cessation of the experiment) since zebrafish can become immobile or swim erratically over the short term period (1 min) but then return to normal when behaviour is assessed over 10 minutes. Supplementary Fig. 3 compares medium timescale (10-minute) detailed FBI, updated every minute, for 2 healthy and 2 treated fish over 3 periods of 30 minutes. For comparative evaluation of behaviour using the method, top level 30 minute FBI was analysed and presented, for example as circled on Fig. 2 (Assessment of each treatment group over the experimental period can be seen in Supplementary Fig. 7).

**Table 2:** When combining the parameters “Distance” and “Activity” from zebrafish behaviour a score is calculated that is normalised to the range 0 to 1, where 1 represents normal pre-treatment or initial behaviour. Based upon the score we assigned categories that can easily be interpreted by human carers from Abnormal, Unhealthy, Ok to Healthy for the Fish Behaviour Index (FBI).

Category	Score	Normalised score
Abnormal	0.0–2.0	0.00–0.33
Unhealthy	2.1–4.0	0.34–0.67
Ok	4.1–5.0	0.68–0.83
Healthy	5.1–6.0	0.84–1.00

FBI analysis was additionally applied to several blinded tests including live testing of the FBI where zebrafish were subject to treatments (e.g. 10% acid, chronic stress) and some additionally given analgesia in real time (Table 3). Time points were selected at random and all 5 points were included for one subject. Qualitative comparisons were made of 10 min FBI with the 30 min indicator and with comments made by an independent observer (with knowledge of zebrafish behaviour) of the videos to validate FBI assessment. Videos of tests with analgesics administered (plus one without) were also assessed blind by a trained observer (trained in behavioural changes associated with invasive procedures) and related to the output of the FBI to verify that automated assessment correlated with human interpretation of zebrafish behaviour.

**Table 3:** Details of live FBI tests where female zebrafish were subject to treatments without (A) and with analgesia (B).

Subject	Time	30 min FBI	10 min FBI	Observer Comments	FBI valid
**A. No analgesic**				**Observer 1**	
Fish 1 Chronic stress	1 (pre)	Healthy	Healthy	No Pain	Y
Fish 2 10%	3	Unhealthy	Abnormal	Pain	Y
Fish 3 10%	1 (pre)	Ok	Ok	No Pain	Y
	2	Unhealthy	Abnormal	Pain	Y
	3	Abnormal	Abnormal	Pain	Y
	4	Abnormal	Abnormal	Pain	Y
	5	Abnormal	Abnormal	Pain	Y
Fish 4 10%	2	Unhealthy	Ok	Pain followed by No pain.	Y
**B. With Analgesic**				**Observer 2**	
Fish 5 FC–8 mgL flunixin	4	Ok	Ok	No pain	Y
Fish 6 FC - Lidocaine 5 mg/L	4	Healthy	Healthy	No pain	Y
Fish 7 FC - Flunixin 1 mg/L	3	Abnormal	Abnormal	Pain	Y
Fish 8 FC - buprenorphine 0.25 mg/L	4	Abnormal	Abnormal	Pain	Y
**A. No analgesic**					
Fish 2 10% (same subject as above)	2	Unhealthy	Abnormal	Pain	Y

Zebrafish were subject to a chronic stress, subcutaneous injection of 10% acetic acid into the frontal lips lip (10%) or fin clip (FC). To validate the FBI assessment the videos were also assessed qualitatively by an independent observer 1 (A) and trained observer 2 (B) blinded to treatment.

### Ethical statement

Experiments were conducted with approval from the Home Office, U.K. (license no. 40/3534) and the University of Liverpool’s Ethics committee. Minimum effective sample sizes were determined using Power analysis. At the end of the experiment after the 6 h behavioural recording, fish were euthanized using a schedule 1 method (concussion followed by brain destruction) and tissue harvested for use in other studies. All fish were treated humanely and care taken when carrying out the treatments. The interventions were chosen as they either represent a standard pain test, such as the acetic acid groups^9,15,29^ or they are routinely used for ID purposes and genomic screening such as the fin clip^40^ (zfin.org/zf_info/zfbook/chapt7/7.8.html) and PIT tagging^41^. The PIT tags used weighed 0.020 g equating to around 2% of the bodyweight of the individuals used in this study which is below the threshold weight of tags known to affect swimming performance^42^. Although there is evidence that benzocaine may be more aversive than metomidate^43^, benzocaine also acts as a local anaesthetic and so provides a period of short-term pain relief pre-operatively thus being the more ethical choice of anaesthetic during these painful treatments. The sham treated group controls for any stress associated with anaesthesia.

## Results

### Post-treatment behavioural changes

**Figure 3:**
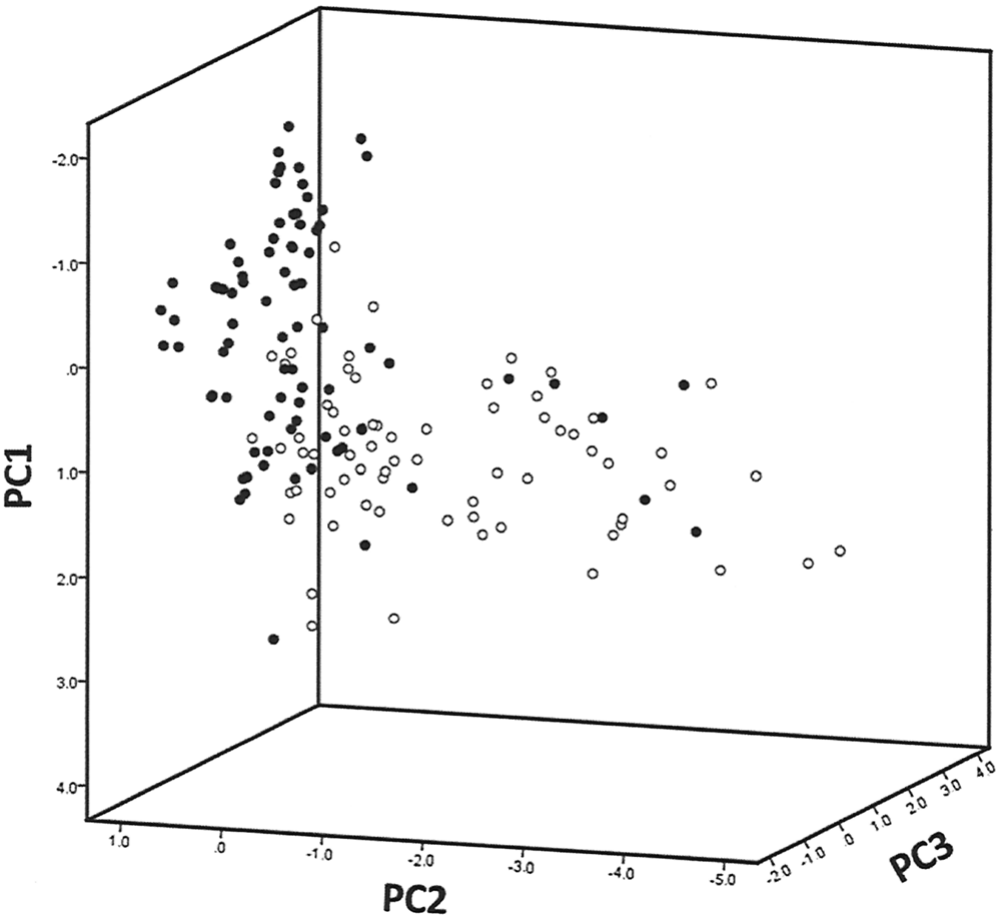
Principal component analysis separating individuals based on the ‘control’ (white spheres) versus ‘pain’ (black spheres) treatment groups. Each data point represents the 20 swim characteristics of an individual (PC1 is average speed, distance; PC2 is % of time in bottom of tank); and PC3 is % tank explored). Supplementary Fig. S8 shows the associated loading plot for PC1 and PC2.

PCA produced 3 components explaining 74% of the variation between control (including sham) and pain groups based on a total of 20 characteristics. The first principal component accounted for 36% of the variation and loaded strongly with the characteristic average speed (distance swam). The second component accounted for 25% of the variation and loaded strongly with the characteristic % of time spent in the bottom of the tank. Thus activity and distance travelled were important behavioural parameters affected by invasive treatment. The 3D plot of the data shows a clear differentiation between the fish from the pain groups, highlighted in black and occupying a vertical plane, and individuals from the control groups, highlighted in white and dispersed across a horizontal plane (Fig. 3; for PCA loading plot for PC1 and PC2 see supplementary Fig. S8).

### Pre-treatment behavior

**Figure 4:**
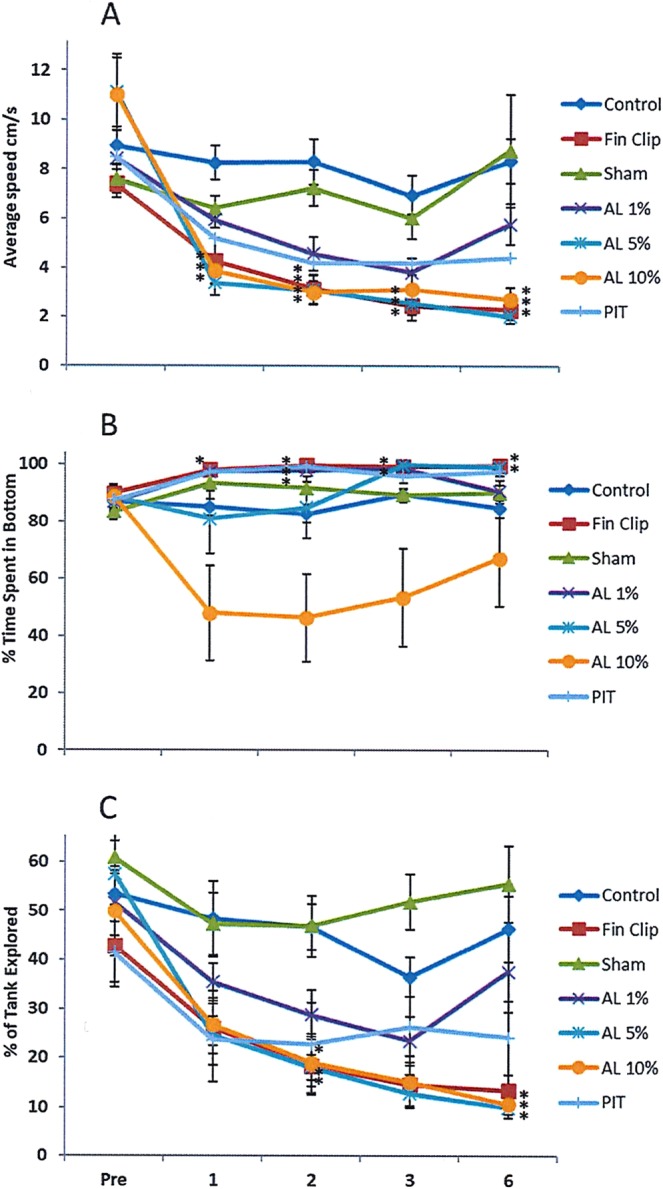
The effect of treatment (control; sham; fin clip (FC); PIT; acid lip (AL) 1%; acid lip (AL) 5%; acid lip (AL) 10%; all groups n = 7) on the behaviour of female zebrafish (mean ± S.E.). Data shown are for average speed (cm/s) (**A**), % time spent in the bottom half of the tank (**B**), and % of tank explored (**C**). *Indicates a significant difference (P < 0.05) when compared to control data.

Prior to any of the treatments there were no differences in average speed or percentage (%) of time spent in the bottom of the tank or % tank explored between any of the groups (P > 0.05; see Supplementary Table 2 for all statistics and Table 4 for post hoc analysis; Fig. 4).

### Speed

A significant reduction in speed relative to controls occurred soon after the procedures in the fin clip, 5% and 10% acid lip groups across the time points 1 (5% acid lip only), 2, 3 and 6 h (P < 0.05). The insertion of a PIT tag also caused a reduction in speed relative to controls but only at the 2 h time point (P < 0.05). The sham handled group did not differ from controls but did have significantly higher speeds than the fin clip, 5% acid lip (2, 3 and 6 h; P < 0.05) and 10% acid lip groups (2 and 6 h; P < 0.05). There was no effect of time on average speed in the control, sham and 1% acid lip groups (P > 0.05) but there was a significant decrease in average speed for fin clip (Pre average speed > 2, 3 and 6 h; P < 0.05), 5% acid lip (Pre > 1, 2, 3 and 6 h; P < 0.05), and 10% acid lip (Pre > 2, 3 and 6 h; P < 0.05). Although there was an effect of time on the PIT group post hoc testing did not detect a difference between pre and post treatment values.

### Tank exploration

Fish within the fin clip and 10% acid lip group exhibited lower % tank explored than controls at 2 and 6 h (P < 0.05; Fig. 4; Supplementary Table 2) with a trend suggesting reductions were also present at 3 h (fin clip P = 0.060; 10% acid lip P = 0.068). A similar pattern was observed in the 5% acid lip group compared with controls but this was only significantly different at 6 h (P < 0.05). Sham handled fish did not differ from controls (P > 0.05) but differ from the fin clip and 10% acid groups at 2, 3 and 6 h (P < 0.05) as well as the 5% acid group at 3 and 6 h (P < 0.05) and PIT tagged fish at 6 h (P < 0.05). Time had a significant effect on % tank explored in the fin clip (Pre > 2, 3 and 6 h; P < 0.05), 1% acid lip (Pre > 2 and 3 h; P < 0.05), 5% acid lip (Pre > 2, 3 and 6 h; P < 0.05) and 10% acid lip groups (Pre > 6 h; P < 0.05). Although a significant effect of time was identified in the PIT tag group (P = 0.033) post hoc tests failed to find a significant difference between time points.

### Time spent at the bottom

Control fish spent less time in the bottom of the tank than the fin clip group at 1, 2, 3 and 6 h (P < 0.05; Fig. 4; Supplementary Table 2), the PIT tag group at 2 h (P < 0.05) the 1% acid lip group at 2 h and the 5% acid lip fish at 3 and 6 h (P < 0.05). Sham handled fish also differed at 3 h from both fin clipped and 5% acid lip fish (P < 0.05). The effect of time was only significant for fin clip (Pre > 1, 2, 3 and 6 h; P < 0.05), PIT (Pre > 1, 2 and 3 h; P < 0.05), 5% acid lip (Pre > 3 h; P < 0.05) and the 1% acid lip group (Pre > 3 h; P < 0.05). The 10% acid lip group displayed a different response to the other treatment groups in that they spent more time at the top of the tank and, therefore, there was no effect of time (Fig. 4).

### Effect of analgesia

There were no significant differences in behaviour between groups prior to fin clip, therefore, administering the drugs prior to the pre-treatment behavioural recording demonstrates the drugs themselves did not alter the behaviour of the female zebrafish (Supplementary Table 3). The analgesic groups were compared with control and fin clip fish without analgesia to determine if behaviour was significantly different from fin clip alone and more similar to control zebrafish behaviour; as such this would signal the analgesic was effective in preventing the fin clip induced behavioural changes.

### Speed

**Figure 5:**
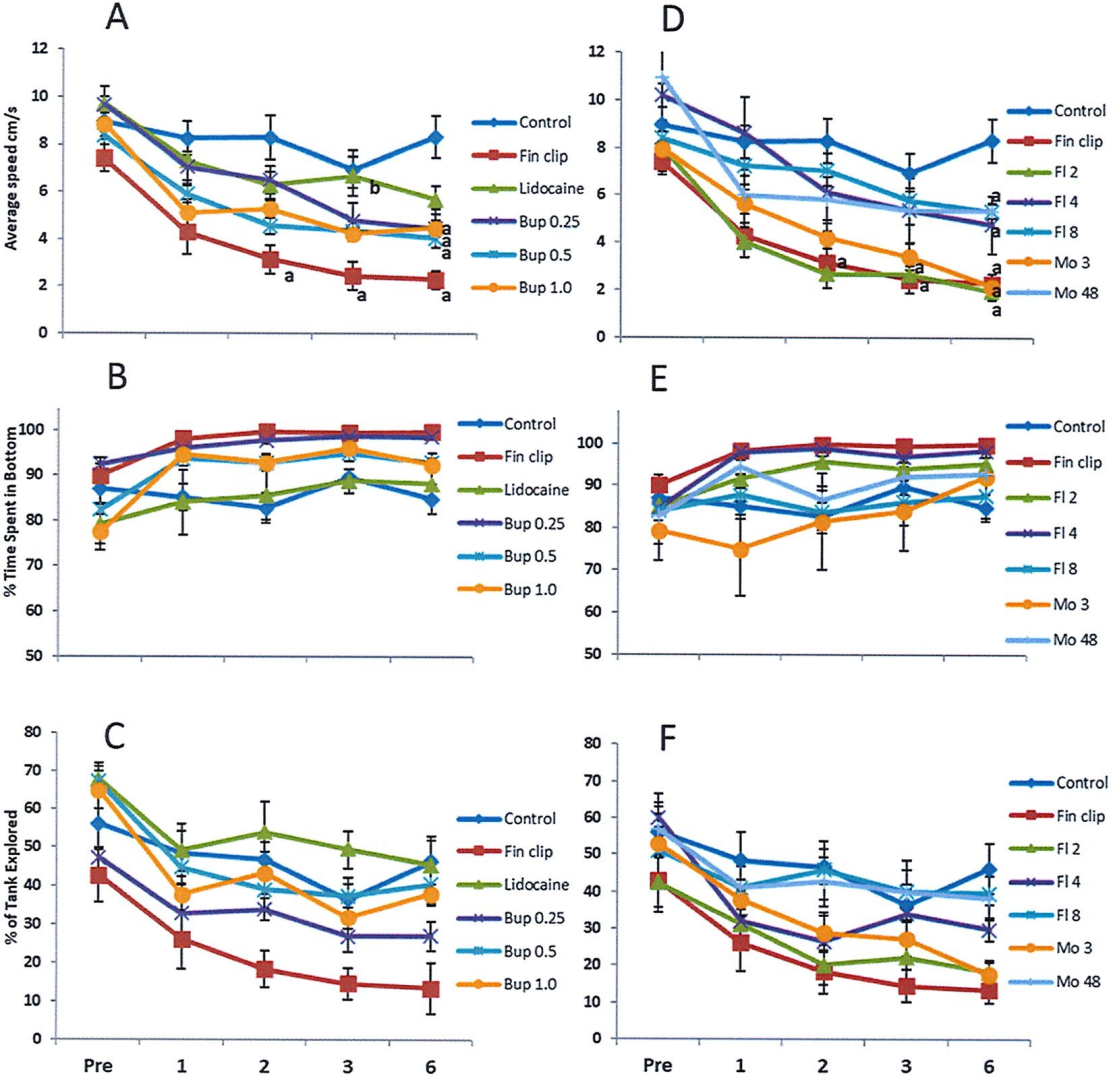
Comparisons between the behaviours (A&D = Average speed (cm/sec^−1^); B&E = % of time spent in the bottom half of the tank; C&F = % of tank explored) of female zebrafish from control and fin clip groups and from fin clipped fish provided with in figs (A–C) the local anaesthetics lidocaine (5 mg/L), 1 mg/L bupivacaine, 0.5 mg/L bupivacaine and 0.25 mg/L bupivacaine and in figs D–F NSAID flunixin at doses 2 mg/L, 4 mg/L, 8 mg/L or the opioid morphine at the doses 3 mg/L and 48 mg/L (n = 6 per dose). A significant difference (P < 0.05) relative to control and fin clip data is signified by the letters ‘a’ and ‘b’ respectively.

The analgesics tested in this study varied considerably in their ability to prevent the reductions in speed observed in the fin clip group (see Supplementary Table 3 for statistics and Table 5 for post hoc analysis). The lowest dose of flunixin (2 mg/L) had the least impact on the fin clip procedure; relative to controls the average speed of this group was significantly reduced at the time points 2, 3 and 6 h (P < 0.05; Fig. 5). Although not quite significant a similar trend was also observed in the lowest dose of morphine (3 mg/L) where speeds appeared to be reduced relative to control at 2 (P = 0.073), 3 (P = 0.089) and 6 h (P < 0.001). The most successful analgesics, however, had speeds that differed from fin clipped fish but not controls. This pattern was observed in the lidocaine group at the time point 3 h (p < 0.05). At 6 h only lidocaine, 8 mg/L flunixin and 48 mg/L morphine had behaviour that was similar to controls (P > 0.05). All other analgesics at 6 h differed from the control group. The effect of time on each treatment group also highlighted the efficacy of the analgesics tested; the most successful analgesics being those who had no significant differences between pre-treatment speed and post hoc testing between each time point: the only group where this was observed was the 48 mg/L morphine group (P > 0.05). Reductions in speed were observed to varying extents in all other analgesic groups; this occurred across 3 time points in 2 mg/L flunixin (pre > 1, 2 and 6 h; P < 0.05), 0.25 mgL bupivacaine and 1 mgL bupivacaine (pre > 2, 3 and 6 h; P < 0.05); 3 time points in 4 mg/L flunixin (1 h > 2, 3 and 6 h; P < 0.05); 2 time points in 3 mg/L morphine and lidocaine (pre > 3 and 6 h; P < 0.05); 1 time point 8 mg/L flunixin (pre > 6 h; P < 0.05) and 0.5 mgL bupivacaine (1 h > 6 h; P < 0.05).

### Tank exploration

In the mixed model analysis there was an effect of time and treatment independently but no interaction. This is likely due to the number of treatment groups in our stringent analysis with many drug doses having intermediate values between control and fin clip groups (Fig. 5; Supplementary Table 3). The effective analgesics resulted in greater % tank explored than that observed in the fin clip group. This trend was observed in the lidocaine and 8 mg/L flunixin groups at 2, 3 and 6 h, in the 0.5 ml/L bupivacaine and 48 mg/L morphine group at 3 and 6 h and the 1 mg/L bupivacaine group (Fig. 5) at 6 h. The lowest dosages of flunixin and morphine failed to ameliorate the impact of the fin clip and resulted in levels of tank exploration that were lower than those observed in controls (Fig. 5; Supplementary Table 3).

### Time spent at the bottom of the tank

Again there was an effect of time and treatment independently but no interaction between the two on time spent at the bottom. The behavioural response to the fin clip was characterised by an increase in the time spent on the bottom of the tank. Zebrafish exposed to the highest dose of bupivacaine (1 mg/L) appeared to spend less time in the bottom of the tank than the fin clip group at 2, 3 and 6 h (Fig. 5; Supplementary Table 3). A similar pattern was observed with 0.5 mg/L bupivacaine at 2 h, lidocaine at 3 and 6 h and 48 mg/L morphine at 2 and 6 h. The other doses of analgesics differed from control but not fin clip groups thus were deemed ineffective.

### The FBI

The FBI was applied to these videos in the present study blind to determine the accuracy for categorizing behaviour without knowledge of treatment. Figure 2 illustrates examples of the FBI output on several videos of different treatments to indicate the relative behaviour of each subject, as compared with their starting state of normal. The FBI successfully gauged the behaviour of the zebrafish in each video even though the FBI operator was blinded to treatment. Control, Sham and pre-treatment behaviour from the pain groups were rated as Normal/Healthy whereas post treatment pain videos were rated as Abnormal/Unhealthy. The ratings range from normal (Healthy) to abnormal over the latest 30 (circled), 20, 10 and 1 minutes, for periods 1 (untreated) and 2, 3 (treated). The PIT fish achieves a low score compared with Control, Sham and Acid Lip (1%). The low score effectively means that this fish was less active and swam shorter distances in response to this treatment compared with the Control, Sham and Acid Lip fish. The categories were labelled in this way to indicate increasing behavioural disruption and increasing concern as scores fall and so human carers could easily determine whether action was necessary. Supplementary information details evaluations of the FBI system (Supplementary Figs 3 and 5), blind testing on other treatments, analgesics (Table 2), and using one camera i.e. 2D analysis (Supplementary Fig. 5). Videos and an image of the system in action are also included in Supplementary Information (Supplementary Table 1 and Fig. 6).

The blind tests covered a wide range of behaviours in real time and highlight that treatments were related to the FBI output (Table 2). The FBI correctly characterized the treatments across the 10 and 30 minute time scales. On the 10 min basis, it would have been correct to have intervened with those zebrafish showing abnormal behaviour since 10 minutes gives an accurate reflection of treatment type and welfare can be improved quickly. There was a direct match between FBI and observers’ comments, indicating that the FBI evaluations were consistent with assessments made by human experts.

## Discussion

Female zebrafish behaviour was profoundly affected by the invasive procedures employed in the present study, characterized as a reduction in swimming speed, the amount of the tank explored and an increase in the use of the bottom of the tank. Indeed the fin clip and 5/10% acid lip groups had not recovered by the end of the 6 h experiment. This prolonged, non-reflexive behavioural response after 6 hours indicates that the response was not a simple nocifensive reflex^28,44^, but was a substantial modification of the animal’s normal behaviour^5^. This response was distinguishable from sham-handled fish whose behaviour did not differ discernibly from controls thus the changes in behaviour were not due to the stress of anaesthesia and handling^28^. The fin clip procedure resulted in a similar change in behaviour to that observed via the insertion of PIT tags and the injection of >5% acetic acid; reductions in average speed, tank exploration and a clear preference for the bottom of the tank were all observed after treatment. Relative to the insertion of PIT tags, however, the fin clip procedure resulted in a significant departure from control behaviour earlier (from 1 h) and across more time points suggesting that the impact of the fin clip was more immediate and potentially of greater severity relative to the PIT tag (see supplementary information for a detailed discussion of the behavioural impacts of different treatments). The changes in behaviour are similar to previous studies in zebrafish employing fin clipping^13,14^. Currently, procedures which result in mild or acute pain for a few hours are deemed to be of mild severity under EU legislation (http://ec.europa.eu/environment/chemicals/lab_animals/pdf/report_ewg.pdf) but our results show that the responses to fin clipping persists for several hours and as such should be deemed moderately severe. However, with the use of immersion analgesia to alleviate any associated pain, this procedure could be reduced to mild. The efficacies of four drugs, from three different classes of analgesics (NSAIDs, opioids and local anaesthetics), differed in their ability to ameliorate the behavioural change induced by the fin clip procedure. Lidocaine (5 mg/L) was the most successful analgesic tested, since it reduced the effect of the fin clip across all three behaviours for the duration of the experiment, resulting in behaviours that consistently aligned with that observed in the control group. Taken together it would seem from the present study that lidocaine (5 mg/L) administered via immersion prior to treatment is the most effective drug to prevent fin clip induced changes. Morphine was also effective but given the cost and regulatory restrictions in its use it may not be widely adopted (see supplementary information for more detailed discussion on the impact of the drugs). We recommend that analgesia is provided for all invasive procedures that cause tissue damage and may give rise to the sensation of pain to ensure good welfare. However, where the analgesic drug itself may confound data collection and justifiably cannot be used then experimenters should wait 24 hours after an invasive procedure before beginning behavioural data collection to allow the zebrafish to recover.

Behavioural responses can be used to determine the internal state of an animal. For example, self-selection of analgesics when subject to a potentially painful event indicates the animal gets pain relief from taking medicated food or water^45^. Facial expression of rodents^46–48^ and of human babies^49^ has led to the development of pain scales where the intensity of pain is linked to the extent of facial changes. However, assessing welfare related behavioural changes in laboratory fish is relatively under-developed compared with mammals and birds^5^ although useful physical terms have been proposed^7,8^. In the context of monitoring fish behaviour other tools have been developed for aquaculture, for example Welfaremeter for a large sea cage (www.imr.no/welfaremeter/about.htm; www.phys.org/news/2012-08-norwegian-produced-fish-welfare-technology-commercial.html) and Seneye (www.seneye.com/what.html) for ornamental fish keeping but these monitor water quality and environmental parameters. Whilst very useful in those contexts, it is unlikely that water quality will change rapidly over ten minutes as we have found that behaviour does after a laboratory procedure^14^ using the FBI. Thus behaviour provides an unobtrusive, quicker and more subtle means of assessing individual status.

Here we demonstrate there are substantial behavioural changes in female zebrafish in response to laboratory procedures that are prevented by the use of a range of pain-relieving drugs at specific doses. This confirms previous work from our laboratory^13,14^ and from other laboratories e.g.^29^. Further drugs with pain-relieving properties given at specific doses prevented the behavioural changes in fin clipped zebrafish confirming recent results on the isolated testing of individual drugs in zebrafish^29^. Our results can help develop effective analgesic protocols for zebrafish in future studies when studying pain is not the objective and further responses to pain may confound the data^1^. Developing an automated monitor, such as the FBI, that reliably and accurately gauges the status of zebrafish behaviour overcomes possible disadvantages of human based assessment including error, bias and constraints on staff time. Applying the FBI which combines two key derived behavioural parameters reliably reflects the treatments experienced by the zebrafish and discriminates stressed or abnormal behaviour from normal healthy (Control) behaviour. FBI can also indicate when behaviour returns to normal. For example, the acid treated fish recover between 3 and 6 hours as seen in previous studies exposing fish to this stimulus^12,15,27,29,32^. Fish, such as zebrafish, goldfish and rainbow trout, significantly reduce activity after a potentially painful stimulus^12,15,28,50,51^ however, some species such a Nile tilapia and piaçu display increased activity^52,53^. Irrespective of the nature of change, the FBI system detects aberration from normal, so either positive or negative changes in baseline behaviour indicate a change in the status of fish, providing scope for testing alterations in fish behaviour performance with a range of experimental paradigms that may inhibit or excite activity (e.g. pharmaceuticals, toxicants) in the future. We used female zebrafish only to prevent gender presenting a confounding effect as has been observed in other behavioural studies^20–25^, however, we propose extending the testing of the FBI to males and to other strains of zebrafish would be valuable since strains may differ in behaviour^54^. In the case of gender differences in zebrafish behaviour, however, studies have shown that male and female behaviour does not differ in response to a variety of painful stimuli and thus it is highly likely that males will show the same responses^29,30^. Since the FBI system represents a generic approach to providing non-intrusive monitoring, combining behavioural characteristics, and evaluating the resultant combination, it may be applied in a wide variety of 3D and 2D behavioural domains.

Other useful behavioural tools (e.g. ID tracker)^55^ do not provide a method of categorizing behaviour thus FBI provides a more meaningful interpretation of the data obtained. Given the laboratory procedures in the present study represent a scale of potentially increasingly noxious stimuli the FBI can determine that mild severity is observed in the Acid Lip (1%) through to higher severity in the Fin Clip. This rating of severity may well match the levels of discomfort that the fish are capable of experiencing under these circumstances when anaesthesia wears off (~15 mins)^31^. The FBI can be used to indicate the actual severity and duration of a variety of procedures since the definition of severity is based upon the degree of behavioural change from normal and our tool provides a means of determining the intensity of such change. Given the difficulties of assessing welfare this is an important step forward and presents a useful refinement if implemented in experimental studies using the zebrafish^3^. Future studies should seek to test this in other strains and in other fish species that have comparable behaviour.

Comparing 10 and 1 min FBI indicators for a healthy fish, 10 minute status appears to reflect current overall status based upon individual behaviour. Fish can appear abnormal over 1 minute if particularly hyper- or hypo-active or excessively sedentary, however, behaviour over 10 minutes is a more meaningful time period to gauge the wellbeing assessment. Animal technicians or researchers can, therefore, be alerted to attend to their experimental subjects in a timely manner to allow them to improve wellbeing possibly by administering analgesia when invasive procedures have been employed^31^. We propose that for ensuring ethical experimentation^1^, pain should be minimised and analgesia administered when practically possible and where it does not confound data collection. FBI also removes human error or bias in behavioural sampling since the software makes a judgement based upon the animal’s behaviour measuring attributes accurately that cannot be done by human eye (e.g. distance swum in 3D or 2D). The adoption of FBI in large scale laboratory fish facilities would reduce staff time spent on welfare checks as well as providing an error free means of measuring fish behaviour. Future studies should extend the FBI’s use to other experimental contexts and to assessing pairs and groups of fish.

Live automated monitoring of female zebrafish welfare has led to the development of the FBI whose utility is in identifying individuals showing unhealthy or abnormal behaviour when exposed to stressors. It can clearly discriminate normal from abnormal welfare in a number of procedures, which would enable alerts to be generated to researchers to intervene to improve welfare within 10 minutes of aberrant behaviour being displayed. Although other useful automated sensors for measuring putative welfare do exist in mammals (e.g. activity monitors in farm animals^56^; commercially available rodent activity monitors e.g. Sense Well (www.noldus.com/projects/sensewell), Rodent Big Brother^57^), these require the animal to carry cumbersome equipment such as collars or have invasive data loggers, microchips or tags which may compromise welfare. Our system allows the monitoring of freely moving fish and again presents a refinement reducing the invasiveness of animal behavioural tracking.

### Data files and software

The datasets and software generated during and/or analysed during the current study are available in the Figshare repository (raw data 10.6084/m9.figshare.7964540; software and source code 10.6084/m9.figshare.7971122 and 10.6084/m9.figshare.7971083; and the FBI at 10.6084/m9.figshare.7991600).

## Supplementary information

Supplementary Info

SI video 1

SI video 2

FM1

FM2

FM3

FM4

FM6

FM7
