# Supplementary material for: Automated monitoring of behaviour in zebrafish after invasive procedures
Source: Sci Rep. 2019 Jun 21;9:9042. doi: 10.1038/s41598-019-45464-w (PMC6588586; doi:10.1038/s41598-019-45464-w)
Supplement: Supplementary file 1 — Supplementary Info [file 41598_2019_45464_MOESM1_ESM.pdf]

Automated monitoring of behaviour in zebrafish after invasive procedures

Anthony G. Deakin[1,2], Jonathan Buckley[2], Hamzah S. AlZu'bi[1], Andrew R. Cossins[3], Joseph W. Spencer[1], Waleed Al’Nuaimy[1], Iain S. Young[3], Jack S. Thomson[4] and Lynne U. Sneddon[2]

[1]Electrical Engineering and Electronics, University of Liverpool, Liverpool L69 3BX, UK. [2]Department of Evolution, Ecology and Behaviour, Institute of Integrative Biology, The BioScience Building, University of Liverpool, L69 7ZB, UK. [3]Department of Functional and Comparative Genomics, Institute of Integrative Biology, The BioScience Building, University of Liverpool, L69 7ZB, UK. [4]School of Environmental Sciences, University of Liverpool, Nicholson Building, Brownlow Street, Liverpool L69 3GP, UK.

Supplementary Information

□

1. Illustration of the Fish Behaviour Index (FBI) system

The FBI takes data from the previous 1 minute interval and refreshes every 1 minute. In our system we currently have analysis time periods of 1, 10 and 30 minutes, which means that on a rolling 1 minute basis the data from the last 1 minute is reported at time period 1 minute, data from the last 10 minutes in the 10 minute period, and data from the previous 30 minutes in the 30 minute period. The data is therefore analysed in the following periods: 1, 2, 3, …, 30 minutes; 1-10, 2-11, 3-12, … 21-30 minutes; 1-30, 2-31, 3-32, …, t to t+29 minutes. Our videos were generally stopped at 25 minutes due to a restriction in our software on the size of video file acquired. In this case, the 30 minute FBI period would use minutes 1-25 only. However, the 30 minute FBI was only used as an indicative overall evaluation (e.g. SI figure S3) because shorter periods of 10 minutes, updated every 1 minute, were adjudged to be representative analysis periods. The system was also tested over periods of 40 minutes at a time. The number, extent and updating frequency of the FBI time periods may be further adapted to suit the particular design of experiments and interventions.

Fig. S1 illustrates both the latest FBI for different time periods (Fig. S1(a)) and the history detail of the 10 min and 1 min FBI (Fig. S1(b)). Fig. S1(a) shows the higher-level overview of FBI as in Fig. 2 of the main paper. Here, the fish is exhibiting normal healthy behavior on the various timescales. At a deeper historical level, the 1 min indicator shown on Fig. S1(b) provides a picture of behavior on a short timescale and shows considerable fluctuations between Normal and Abnormal FBI. The two Abnormal FBI states in this 40 min period are circled on Fig. S1(b). It was observed that the healthy fish unexpectedly had 2 quiescent periods at minutes 33 and 36 (prolonged periods of resting on the tank bottom) which would be more expected from unhealthy fish. By contrast the 10 min FBI remains constant and at maximal rating of Healthy for this fish after the first 12 minutes.

At an even deeper level, Fig. S2 below illustrates for a Control fish and a PIT tagged fish, displaying the extremes of the scale or index, the ‘raw’ data for their Activity and Distance parameters. Fig. 2 (a) and (d) (main paper) shows the FBI results derived from such underlying raw data of Fig S2.

The FBI analysis module is currently contained in a separate Microsoft Excel file for ease of use and can provide real-time functionality or alternatively be used to analyze subjects' test files (coordinate logs) retrospectively (post processing). It generates the FBI over periods of the latest 1 minute, 10 minutes, 20 minutes and 30 minutes (with provision also for latest 60 minutes). The FBI is qualitative (with categories Healthy, Ok, Unhealthy, Abnormal) and is currently based on a combination of Distance Swum (DS) and Activity (A).

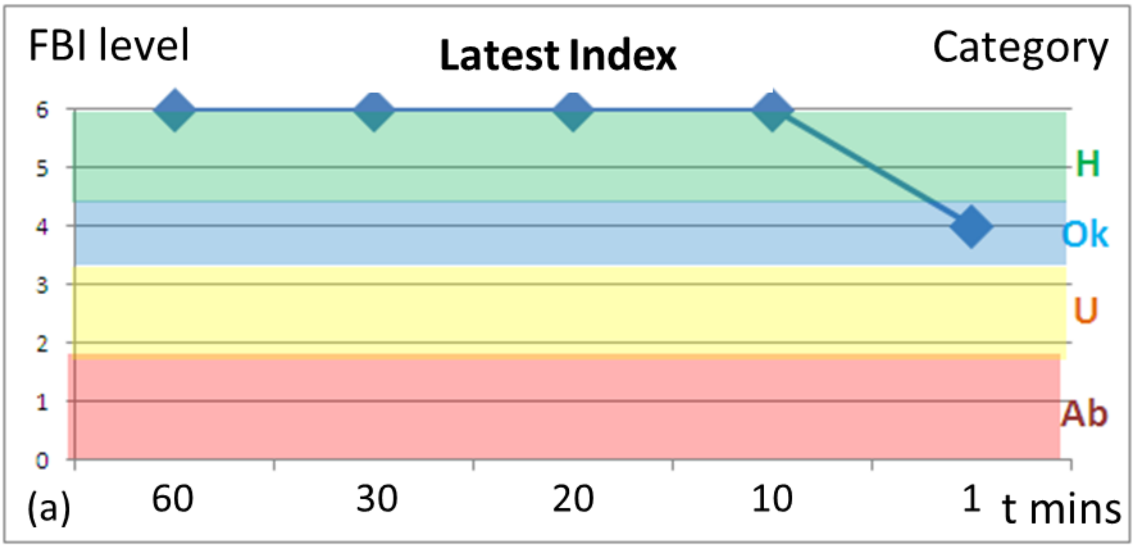

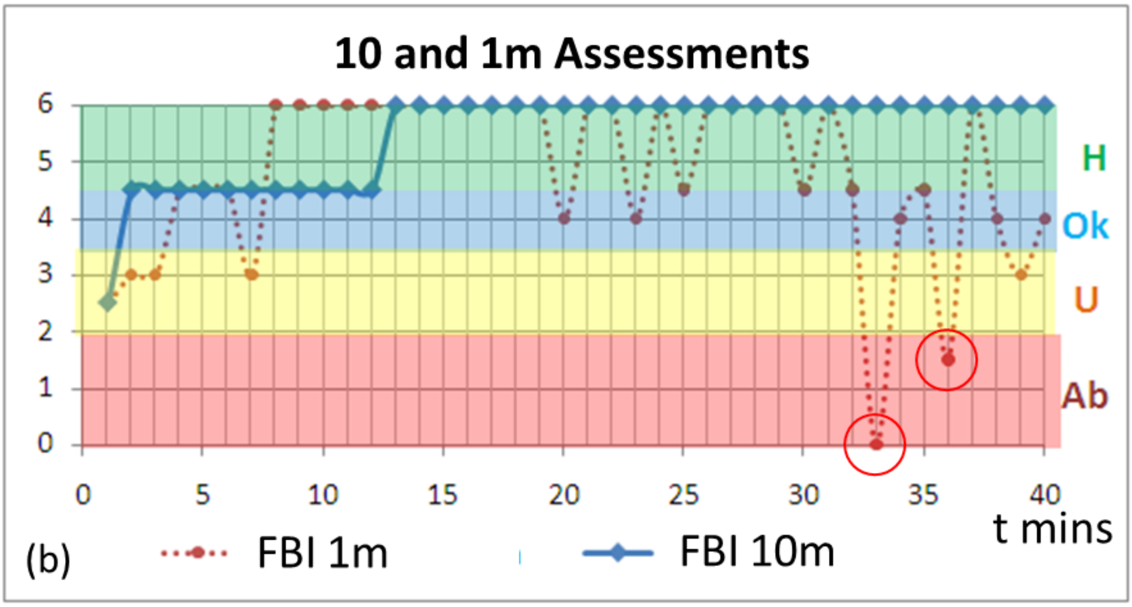

Fig. S1. FBI for a healthy female zebrafish over 40 minutes: (a) after 40 minutes for all timescales (periods of the latest 60, 30, 20, 10, 1 minutes, 60 denotes ≥ 30 etc.) and (b) history over 40 minutes for the 10 min and 1

min indicators, with potentially Abnormal (Ab) behavior circled. FBI is expressed numerically (0 – 6) and categorically (H = healthy, Ok = acceptable, U = unhealthy, Ab = abnormal).

Basis of the Wellbeing index

Fig. S2 illustrates for two zebrafish the raw data of derived A and DS behavioral parameters for the 3D views of the tank (front and top), both over time and as scatter plots. FBI (Figs. S1 and 2 (main)) is based on combining A and DS.

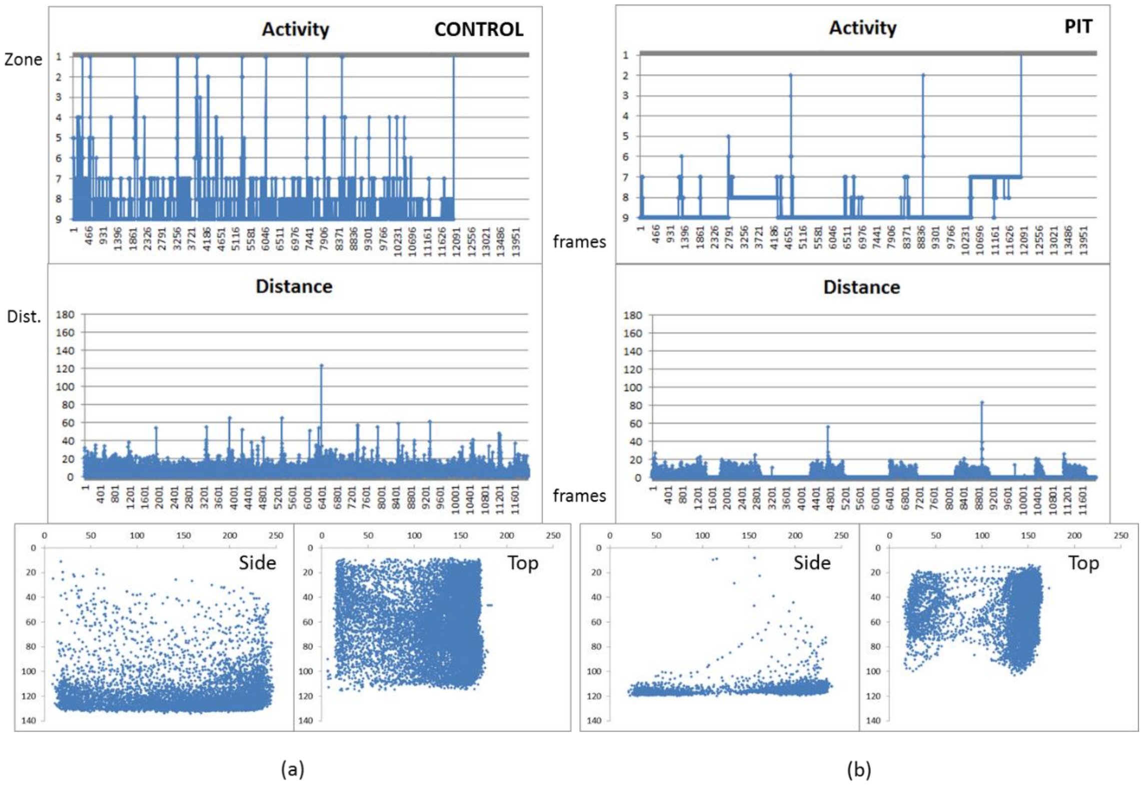

Fig. S2. Views of raw Activity data and Distance, plus front (side) and top views of the location coordinate trajectories after treatment (period 3) for (a) the Control and (b) the PIT treated female zebrafish from Fig. 2 (main) over 25 minutes.

Fig. S2 shows raw Activity as zones (1-9) visited over time (with the tank divided into 9 zones covering the area available to the fish) and Distance travelled over time for the Control and for the PIT fish of Fig. 2 over 25 minutes at post-treatment time point (3). It also shows the front (side) and top views of the tank using plots of the locations visited by the fish. These data are used in the derivation of the FBI of Fig. 2 (main). The raw data of Fig. S2(b) shows marked differences between a PIT treated fish and the Control fish. Fig. S2(b) shows typical characteristics of abnormal wellbeing: very low raw Activity, low changes in raw Activity level; low Distance swum with several periods of stationary behavior between ‘pulses’ of movement; low utilization/exploration of the tank.

2. Evaluation of the system

The deeper system components, 10-minute FBI, for two Control (C) and two Fin Clip (FC) fish are compared on Fig. S3.

10m FBI	C2			C8			FC6			FC2		
period / minute	i	ii	iii	i	ii	iii	i	ii	iii	i	ii	iii
1	Healthy	Ok	Healthy	Healthy	Ok	Healthy	Healthy	Unhealthy	Unhealthy	Ok	Abnormal	Abnormal
2	Healthy	Healthy	Healthy	Ok	Unhealthy	Healthy	Healthy	Unhealthy	Unhealthy	Ok	Abnormal	Abnormal
3	Ok	Healthy	Healthy	Ok	Unhealthy	Healthy	Healthy	Unhealthy	Ok	Ok	Abnormal	Abnormal
4	Healthy	Healthy	Healthy	Ok	Unhealthy	Healthy	Healthy	Unhealthy	Ok	Ok	Abnormal	Abnormal
5	Healthy	Healthy	Healthy	Ok	Abnormal	Healthy	Healthy	Unhealthy	Unhealthy	Ok	Abnormal	Abnormal
6	Healthy	Healthy	Healthy	Healthy	Abnormal	Healthy	Healthy	Unhealthy	Unhealthy	Ok	Abnormal	Abnormal
7	Healthy	Healthy	Healthy	Healthy	Abnormal	Healthy	Healthy	Unhealthy	Unhealthy	Healthy	Abnormal	Abnormal
8	Healthy	Healthy	Healthy	Healthy	Abnormal	Healthy	Healthy	Unhealthy	Unhealthy	Healthy	Abnormal	Abnormal
9	Healthy	Healthy	Healthy	Healthy	Unhealthy	Healthy	Healthy	Unhealthy	Unhealthy	Ok	Abnormal	Abnormal
10	Healthy	Healthy	Healthy	Healthy	Unhealthy	Healthy	Healthy	Unhealthy	Unhealthy	Healthy	Abnormal	Abnormal
11	Healthy	Healthy	Healthy	Healthy	Ok	Healthy	Healthy	Unhealthy	Unhealthy	Healthy	Abnormal	Abnormal
12	Healthy	Healthy	Healthy	Healthy	Ok	Healthy	Healthy	Unhealthy	Unhealthy	Healthy	Abnormal	Abnormal
13	Healthy	Healthy	Healthy	Healthy	Ok	Healthy	Healthy	Unhealthy	Unhealthy	Healthy	Abnormal	Abnormal
14	Healthy	Healthy	Healthy	Healthy	Ok	Healthy	Healthy	Unhealthy	Unhealthy	Healthy	Abnormal	Abnormal
15	Healthy	Healthy	Healthy	Healthy	Ok	Healthy	Healthy	Unhealthy	Unhealthy	Healthy	Abnormal	Abnormal
16	Healthy	Healthy	Healthy	Healthy	Ok	Healthy	Healthy	Unhealthy	Unhealthy	Healthy	Abnormal	Abnormal
17	Healthy	Healthy	Healthy	Healthy	Ok	Ok	Healthy	Unhealthy	Unhealthy	Healthy	Abnormal	Abnormal
18	Healthy	Healthy	Healthy	Healthy	Ok	Ok	Healthy	Ok	Unhealthy	Healthy	Abnormal	Abnormal
19	Healthy	Healthy	Healthy	Healthy	Ok	Ok	Healthy	Healthy	Unhealthy	Healthy	Abnormal	Abnormal
20	Healthy	Healthy	Healthy	Healthy	Ok	Ok	Healthy	Healthy	Abnormal	Healthy	Abnormal	Abnormal
21	Healthy	Healthy	Healthy	Healthy	Ok	Ok	Healthy	Healthy	Abnormal	Healthy	Abnormal	Abnormal
22	Healthy	Healthy	Healthy	Healthy	Ok	Ok	Healthy	Healthy	Abnormal	Healthy	Abnormal	Abnormal
23	Healthy	Healthy	Healthy	Healthy	Ok	Ok	Healthy	Healthy	Unhealthy	Healthy	Abnormal	Abnormal
24	Healthy	Healthy	Healthy	Healthy	Ok	Healthy	Healthy	Healthy	Unhealthy	Healthy	Abnormal	Abnormal
25	Healthy	Healthy	Healthy	Healthy	Ok	Healthy	Healthy	Healthy	Ok	Healthy	Abnormal	Abnormal
30m FBI	H	H	H	H	O	H	H	O	U	H	A	A
	1	(a)		2			1	(b)		2		

Fig. S3. Rolling 10-minute FBI plus overall 30-minute FBI for 4 fish over 25 minutes each. Examples (a)(1)(2) Two Control fish (b)(1)(2) Two Fin Clip fish. FBI categories (Healthy (H), Ok (O), Unhealthy (U), Abnormal (A) before (i) and after (ii, iii) treatment for 4 individual fish from the Control and Fin Clip groups over ~ 3 hours in total, for 25 minutes in each period, on the latest 10 minute basis, updated minute by minute. Overall FBI based on the latest 30 minutes is given in capital letters (e.g. HHH) for comparison with the latest 10 minutes. All 4 examples have an initial pre-treatment 30 minute FBI of Healthy.

Fig. S3 shows, over 3 periods (i, ii, iii) of 25 minutes for each of 4 female zebrafish examples (C2, C8, FC2, FC6 chosen at random), their 10-minute wellbeing indices (FBI) using the four categories of wellbeing in declining order (Healthy, Ok, Unhealthy, Abnormal), updated every minute. C2 and C8 belong to the Control group (a) and FC2 and FC6 to the Fin Clip group (b). Period (i) is 40 minutes pre-intervention, periods (ii) and (iii) are 1h and 2h post-procedure. Also shown are the 30-minute wellbeing indices, 30 min FBI, representing the full 25 minutes in each case. All 4 fish were seen to be Healthy (H) pre-intervention (i) on the 30-minute scale. C2 remained healthy throughout whereas C8 has a dip in FBI to ‘O’ in period (ii) before returning to normal H in period (iii). Fin clip FC6 responded similarly to C8 in period (ii) overall (30-minute FBI = O) but then deteriorated further in period (iii) to U. FC2 deteriorates rapidly to unchanging, worst category FBI = A in periods (ii) and (iii).

The 10-minute FBI gives considerable detail on developments in welfare. In period (ii), C8 declined in its normal activity, going through an ‘Ok’ phase which declined further to Unhealthy and Abnormal for 3 and 4 minutes respectively before recovering through U and O for 2 and 15 minutes respectively. In period (iii) it was again Healthy overall, as expected for an untreated fish. The effect of the fin clip on FC2 was dramatic as the fish immediately went into Abnormal condition throughout periods (ii) and (iii) without recovering. By contrast, FC6 declined over periods (ii) and (iii) but only reached Abnormal for 3 minutes. Otherwise, the fish recovered to Healthy for 7 minutes in period (ii). Overall, it appeared less affected by the treatment than the severely affected FC2.

The 10-minute FBI for all five fish of Fig. 2 (main) are compared on Fig. S4. The 10min FBI characterizes more gradual variation in behavior over the medium-short term, whereas the longer term 30 min view was far less sensitive to short term fluctuations.

	(a) Control2			(b) Sham1			(c) AL1			(d) PIT1			(e) FC2		
period / minute	i	ii	iii	i	ii	iii	i	ii	iii	i	ii	iii	i	ii	iii
1	Healthy	Ok	Healthy	Healthy	Healthy	Healthy	Unhealthy	Ok	Unhealthy	Healthy	Ok	Unhealthy	Ok	Abnormal	Abnormal
2	Healthy	Healthy	Healthy	Healthy	Healthy	Healthy	Unhealthy	Ok	Unhealthy	Healthy	Abnormal	Abnormal	Ok	Abnormal	Abnormal
3	Ok	Healthy	Healthy	Healthy	Healthy	Ok	Ok	Unhealthy	Unhealthy	Healthy	Abnormal	Abnormal	Ok	Abnormal	Abnormal
4	Healthy	Healthy	Healthy	Healthy	Healthy	Ok	Ok	Unhealthy	Unhealthy	Healthy	Abnormal	Abnormal	Ok	Abnormal	Abnormal
5	Healthy	Healthy	Healthy	Healthy	Healthy	Ok	Ok	Ok	Unhealthy	Healthy	Abnormal	Abnormal	Ok	Abnormal	Abnormal
6	Healthy	Healthy	Healthy	Healthy	Healthy	Ok	Ok	Unhealthy	Abnormal	Healthy	Abnormal	Abnormal	Ok	Abnormal	Abnormal
7	Healthy	Healthy	Healthy	Healthy	Healthy	Ok	Ok	Ok	Abnormal	Healthy	Abnormal	Abnormal	Healthy	Abnormal	Abnormal
8	Healthy	Healthy	Healthy	Healthy	Healthy	Ok	Ok	Ok	Abnormal	Healthy	Abnormal	Abnormal	Healthy	Abnormal	Abnormal
9	Healthy	Healthy	Healthy	Healthy	Healthy	Ok	Ok	Ok	Abnormal	Healthy	Abnormal	Abnormal	Ok	Abnormal	Abnormal
10	Healthy	Healthy	Healthy	Healthy	Healthy	Ok	Ok	Ok	Abnormal	Healthy	Abnormal	Abnormal	Healthy	Abnormal	Abnormal
11	Healthy	Healthy	Healthy	Healthy	Healthy	Ok	Ok	Ok	Abnormal	Healthy	Abnormal	Abnormal	Healthy	Abnormal	Abnormal
12	Healthy	Healthy	Healthy	Healthy	Healthy	Ok	Ok	Ok	Abnormal	Healthy	Abnormal	Abnormal	Healthy	Abnormal	Abnormal
13	Healthy	Healthy	Healthy	Healthy	Healthy	Ok	Ok	Ok	Abnormal	Healthy	Abnormal	Abnormal	Healthy	Abnormal	Abnormal
14	Healthy	Healthy	Healthy	Healthy	Healthy	Ok	Ok	Ok	Abnormal	Healthy	Abnormal	Abnormal	Healthy	Abnormal	Abnormal
15	Healthy	Healthy	Healthy	Healthy	Healthy	Healthy	Ok	Ok	Abnormal	Healthy	Abnormal	Abnormal	Healthy	Abnormal	Abnormal
16	Healthy	Healthy	Healthy	Healthy	Healthy	Healthy	Healthy	Ok	Abnormal	Healthy	Abnormal	Abnormal	Healthy	Abnormal	Abnormal
17	Healthy	Healthy	Healthy	Ok	Healthy	Healthy	Healthy	Ok	Abnormal	Healthy	Abnormal	Abnormal	Healthy	Abnormal	Abnormal
18	Healthy	Healthy	Healthy	Ok	Healthy	Healthy	Healthy	Ok	Abnormal	Healthy	Abnormal	Abnormal	Healthy	Abnormal	Abnormal
19	Healthy	Healthy	Healthy	Ok	Healthy	Healthy	Healthy	Ok	Unhealthy	Healthy	Abnormal	Abnormal	Healthy	Abnormal	Abnormal
20	Healthy	Healthy	Healthy	Healthy	Healthy	Healthy	Healthy	Ok	Unhealthy	Healthy	Abnormal	Abnormal	Healthy	Abnormal	Abnormal
21	Healthy	Healthy	Healthy	Healthy	Healthy	Healthy	Healthy	Ok	Unhealthy	Healthy	Abnormal	Abnormal	Healthy	Abnormal	Abnormal
22	Healthy	Healthy	Healthy	Ok	Healthy	Healthy	Healthy	Ok	Unhealthy	Healthy	Abnormal	Abnormal	Healthy	Abnormal	Abnormal
23	Healthy	Healthy	Healthy	Ok	Healthy	Healthy	Healthy	Ok	Ok	Healthy	Abnormal	Abnormal	Healthy	Abnormal	Abnormal
24	Healthy	Healthy	Healthy	Ok	Healthy	Healthy	Healthy	Ok	Ok	Healthy	Abnormal	Abnormal	Healthy	Abnormal	Abnormal
25	Healthy	Healthy	Healthy	Ok	Healthy	Healthy	Healthy	Ok	Unhealthy	Healthy	Abnormal	Abnormal	Healthy	Abnormal	Abnormal

Fig. S4. Fish Behaviour Index (FBI) categories (Healthy, Ok, Unhealthy, Abnormal before (i) and 2 and 3 h after (ii, iii) treatment for 5 individual female fish from the 5 groups (cf Fig. 2 (main)): (a) Control (b) Sham (c) Acid Lip (d) PIT (e) Fin Clip treated zebrafish over ~ 3 hours in total, for 25 minutes in each period, on the latest 10 min FBI basis, updated minute by minute. All 5 examples have an initial pre-treatment 30min FBI of Healthy, which is maintained by Control and Sham in periods (ii) and (iii) but deteriorates to various extents with the other fish.

Fig. S4 shows 10min FBI updated minute by minute. It further illustrates the interplay between 30min and 10min FBI for several behaviors. Sham1 (Fig. S4(b)), has a ‘quieter’ period of 12 minutes in period (iii) seen in the consecutive 10min FBI ‘Ok’ categorizers

extending from minute 3 to minute 14. Nevertheless, overall (25min) it is adjudged Healthy. AL1 (Fig. S4(c)) has similar dips in period (i), which include Unhealthy and Ok but is overall Healthy. In period (iii) it is overall Unhealthy (but not worse than that) after dipping to Abnormal for 13 minutes extending from minute 6 to minute 18. The 10min FBI characterizes more gradual variation in behavior over the medium-short term. The longer term 30 minute view is far less sensitive to short term fluctuations. (An extended version of Figure S4 is available at Figshare (DOI: 10.6084/m9.figshare.7991600) comparing 10min FBI and 30 min FBI).

3. Validation in Two Dimensions

FBI dependence on 3D versus 2D monitoring was investigated since many laboratories may not have the capacity to put video cameras above tanks in a typical zebrafish racking system. This affected distance travelled thus the software was modified to operate with one camera (side view) and FBI was adapted (publically demonstrated at the Blue Planet Aquarium 2015 (www.blueplanetaquarium.com/fish-health-monitor-trial-this-saturday-at-blue-planet). To compare 2D with 3D FBI, the 3D coordinates for the 5 fish of Fig. 2 (main) were also run through the modified 2D FBI system (i.e. 2D FBI utilizing front camera coordinates only). Fifteen videos from experiment 1 were chosen at random and re-assessed using only 2D data. Out of 15 only 2 differed from 3D analysis but were within the Healthy/OK or within Unhealthy/Abnormal categories thus the overall wellbeing status was within healthy and unhealthy states. No videos were outside more than 2 adjacent categories. The results of applying 2D FBI to the fish from the different treatment groups are shown on Fig. S5. This is directly comparable with the 3D FBI illustrated on Fig. 2 (main).

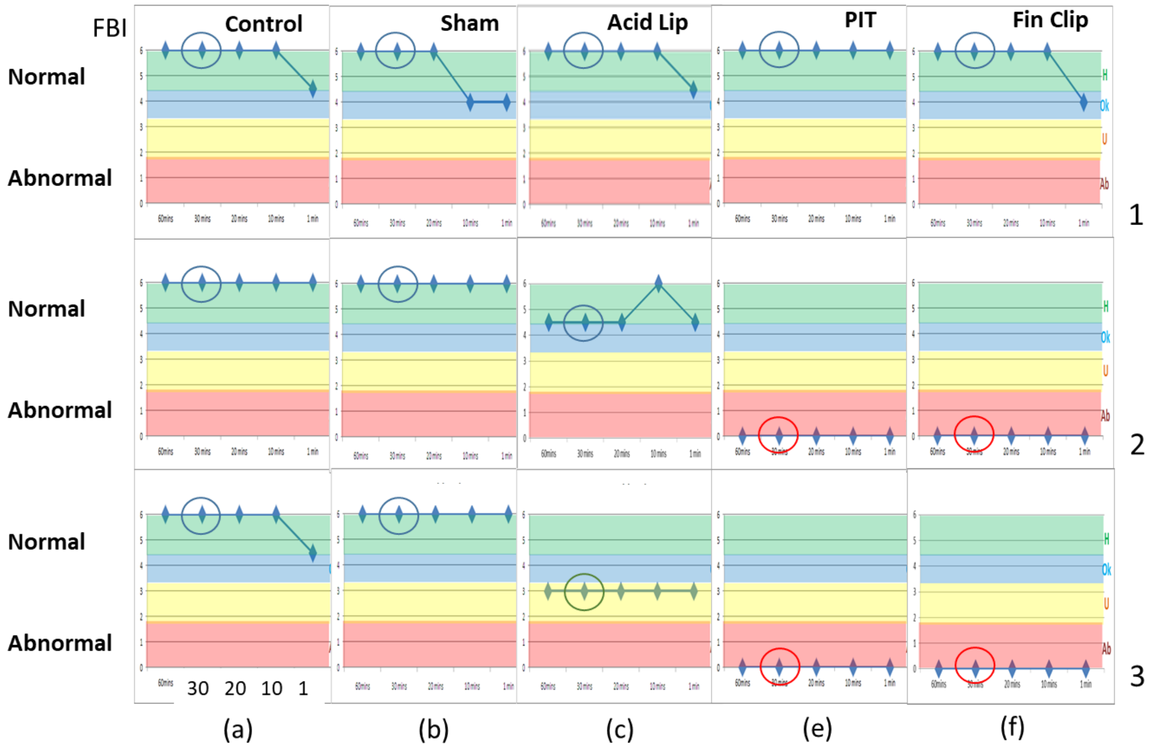

Fig. S5. 2D wellbeing index (0 to 6) before (1) and after (2, 3) treatment for 5 individual female zebrafish from the 5 groups: (a) Control (b) Sham (c) Acid Lip (d) PIT (e) Fin Clip treated zebrafish over ~ 3 hours in total, using 4 timescales each, from the latest 30 mins (circled) to the latest 20 mins, 10 mins and 1 min. Gradation of score from green (Normal/Healthy), blue (Ok/Healthy), yellow (Unhealthy) to pink (Abnormal) provides a means of assessing how the subject's behavior differs from normal and provides a scale to base welfare decision upon.

Only two of 15 results were different between 2D and 3D: Acid Lip time 2 (10-min Healthy instead of Ok); Fin Clip time 3 (1-min Abnormal instead of Unhealthy). Thus this demonstrates the utility of a 2D application of the FBI since the categorical differences are still within Healthy/Ok or Unhealthy/Abnormal and diverge from 3D in this respect in only 13% of videos tested.

4. Equipment Setup and Operation

The system runs tracking software[15] written in Matlab TM which identifies the subject in the tank, optionally records video of the subject for post analysis, and provides the subject's x,y coordinate pairs for each camera for each frame. The cameras are currently run at a pixel resolution of 800 x 600 and a frame rate of 8fps for the selected time period, which is generally 30 minutes, and the subject's coordinates are logged to text file. The software provides a color-coded simulated 3D graphic on the user interface displaying the subject's current location, and recent as well as complete trajectory to date (SI Video 1).

The FB Index and related analyses are provided by the FBI analysis module which can be run in parallel with the tracking.

The FBI module is available on Figshare(DOI: 10.6084/m9.figshare.7991600).

5. Videos of system in use

A number of short videos of the system in use with two untreated fish are also provided in Supplementary Information FM1-3, FM4-7. These show short excerpts from continuous monitoring and illustrate the kinds of short-term behavioral fluctuations, including unhealthy and abnormal on a 1 minute scale, that may naturally occur within normal healthy overall behavior as well as identifying what has caused a change in status (e.g. low distance swum). Six are detailed in the following Table 1 and an example screen of the system in use is shown in Fig. S6. Additional videos include the tracking system in operation and an example of a fish improving to Healthy from Unhealthy (SI Videos 1 and 2).

Table 1: Details of video examples of the system screen in operation for 2 untreated (healthy) female zebrafish (with updates every 1 min). H (healthy), O (Ok), U (Unhealthy), A (Abnormal).

Fish	Video Ref	Period covered (mins)		Wellbeing over latest period (mins)			10min basis Comments	Reason	1min basis Comments
		start	end	20	10	1			
1	FM1	0	02:30	H	H	U	Healthy reduces to	low dist.	Unhealthy deteriorates to
1	FM2	02:30	03:30	O	O	A	Ok stays		Abnormal improves to
1	FM3	04:30	05:30	O	O	U	Ok	(low dist.)	Unhealthy
Overall for this timescale: seems Ok, slightly low energy, with some short-term fluctuations.									
2	FM4	0	04:45	H	H	O	Healthy Stays		OK improves to

2	FM6	09:45	09:45	H	H	H	Healthy reduces to		Healthy stays
2	FM7	16:45	16:45	H	O	H	Ok	low activity	Healthy
Overall for this timescale: seems Normal, intermittently exploring full tank volume.									

The following screen example (Fig. S6) shows the fish subject (right, identified with pink circles), its trajectory (center right) and the detailed FBI analyses (left half) and figure S7 demonstrates how the FBI assesses zebrafish from each treatment group 2 h after treatment.

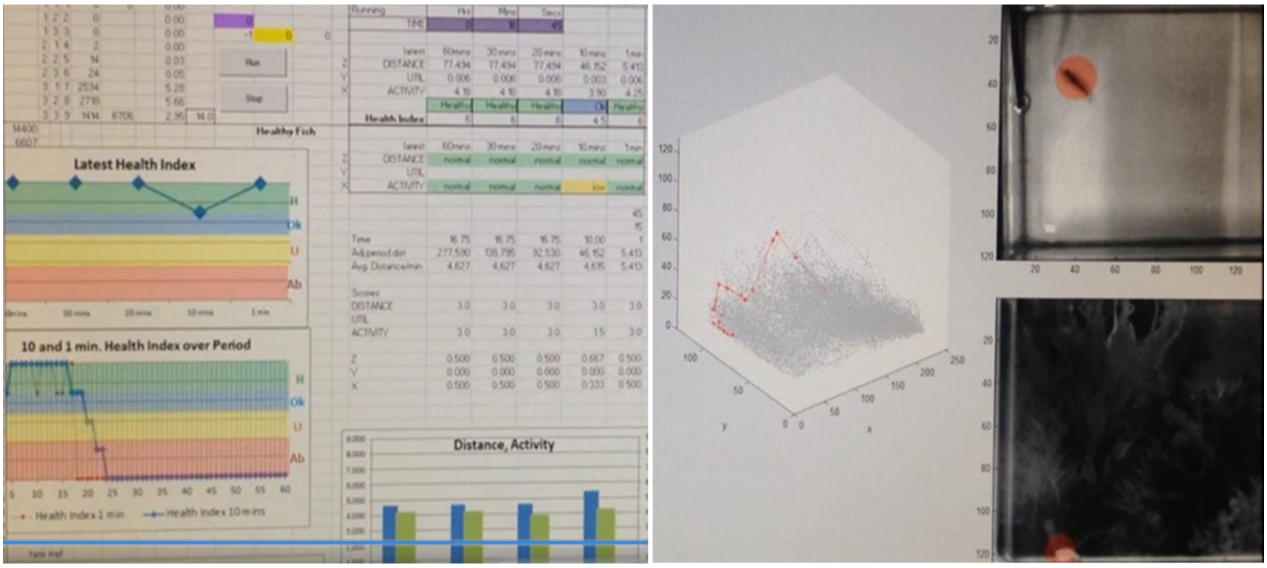

Fig. S6: Screen example showing the subject fish (right), trajectory (center right), FBI (left).

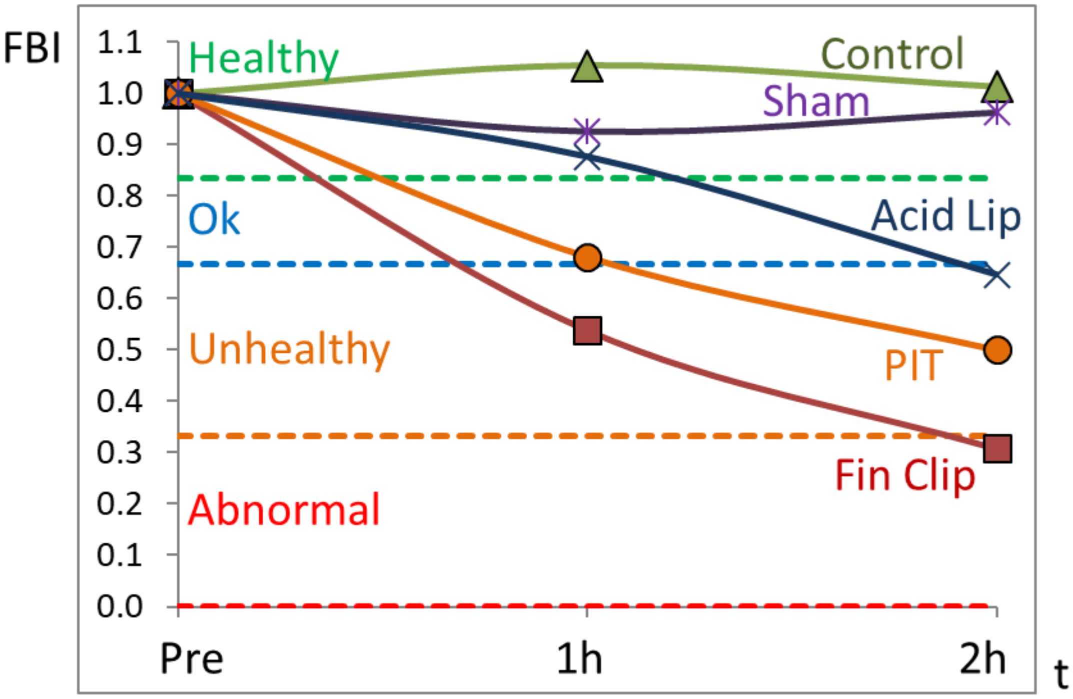

Fig. S7. Summary FBI analysis of videos taken before treatments and at 1 h and 2 h afterwards for the zebrafish from Control, Sham, Acid Lip (1% acetic acid), Pit Tagged and Fin Clip groups demonstrating that Control and Sham fish remain normal, Fin clip become Abnormal and other treatments are assessed as unhealthy.

Fig S7 presents, for all the subjects in the 5 groups of female zebrafish, mean FBI for the 30 minute timescale at the 3 initial timepoints (pre, 1h, 2h). For clarity, each group is normalized to its mean Pre value. FBI of the Control and Sham groups remained virtually constant and Healthy throughout. FBI for the Fin Clip group, which is most affected, declined by ~50% at timepoint 2 and another ~25% from timepoint 2 to 3, taking this group into the Abnormal FBI

category. The other treated groups responded similarly to Fin Clip but with reduced severity. PIT ended at timepoint 3 with FBI ~50% reduced from pre-treatment whereas the least affected group, Acid Lip, has FBI reduced by about 35%. Acid Lip and PIT treated fish reached the Unhealthy category but avoided the Abnormal category. The results from the FBI in Figure S7 reflect the statistically significant results obtained for the Acid Lip 1%, PIT and Fin Clip treatment groups (Table S2) based on individual parameters from the Principle Components Analysis (Figs. 4 and 5).

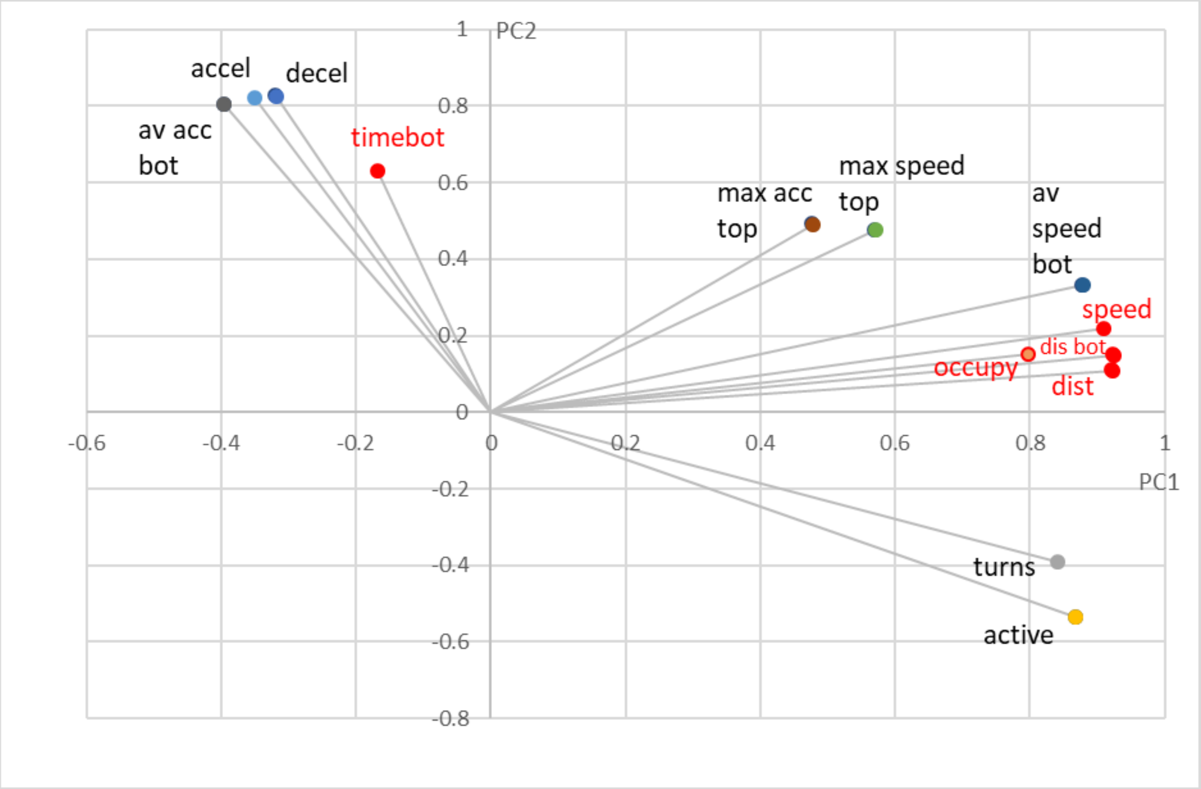

Fig. S8. PCA loading plot for PC1 and PC2 showing the resulting 13 main behavioural characteristics, as included within Table 1. Major characteristics are shown in red.

Fig S8 shows the loading plot for PC1 and PC2 of the PCA analysis (Fig. 3) which informed the design of the FBI.

6. Statistical Analysis of Behavioural Data

Table 2. The impact of potentially painful procedures (Treatment) on the behaviour of female zebrafish. (A) Mixed Model Analysis exploring effects of Treatment (Control, Sham, Fin Clip, PIT tagged, and 1, 5 and 10% acetic acid), Time (pre-treatment, 1, 2, 3 and 6h after treatment; n = 7 per group) and their interaction. Post-hoc analysis of the effect of Time on behaviour for different Treatments (B) and the effect of Treatment on behaviour at different times (C; d.f. for all terms = 6,42) are also presented. Analyses include Huynh-Feldt (*) or Greenhouse-Geisser (†) corrections for sphericity as appropriate. Degrees of freedom presented as: term, error.

(A)

**Swim Characteristics**	**Model Term**	**Degrees of Freedom**		***F***	***p***	
Average Speed†	Time × Treatment	14.5,	122.3	4.08	< 0.0005	***
	Time	2.9,	122.3	49.81	< 0.0005	***
	Treatment	6,	42	5.76	< 0.0005	***
% of time spent in the bottom of the tank*	Time × Treatment	23.2,	162.6	3.10	< 0.0005	***
	Time	3.9,	162.6	10.92	< 0.0005	***
	Treatment	6,	42	7.30	< 0.0005	***
% Tank Explored†	Time × Treatment	16.7,	116.7	2.91	< 0.0005	***
	Time	2.8,	116.7	43.88	< 0.0005	***
	Treatment	6,	42	5.80	< 0.0005	***

(B)

**Swim Characteristics**	**Treatment**	**Degrees of Freedom**		***F***	***p***	
Average Speed	Control	4.0,	24.0	1.48	0.241	
	Fin Clip†	1.4,	8.4	20.42	0.001	**
	Sham†	1.4,	8.1	0.83	0.425	

	Pit Tag	4.0, 24.0	7.30	0.001	**
	Acid 1%†	1.6, 9.4	3.11	0.099	
	Acid 5%	4.0, 24.0	54.76	< 0.0005	***
	Acid 10%	4.0, 24.0	12.98	< 0.0005	***
% of time spent in the bottom of the tank	Control	4.0, 24.0	0.91	0.474	
	Sham	4.0, 24.0	2.75	0.052	
	Fin Clip†	2.0, 11.7	38.24	< 0.0005	***
	Pit Tag	4.0, 24.0	9.74	< 0.0005	***
	Acid 1%	4.0, 24.0	6.27	0.001	**
	Acid 5%†	1.4, 15.0	4.54	0.055	
	Acid 10%	4.0, 24.0	0.87	0.494	
% Tank explored†	Control	4.0, 24.0	1.69	0.185	
	Sham	4.0, 24.0	1.98	0.130	
	Fin Clip	4.0, 24.0	18.64	< 0.0005	***
	Pit Tag†	2.1, 12.4	3.47	0.062	
	Acid 1%	4.0, 24.0	10.47	< 0.0005	***
	Acid 5%	4.0, 24.0	25.36	< 0.0005	***
	Acid 10%	4.0, 24.0	7.67	< 0.0005	***

(C)

**Swim Characteristics**	**Time**	***F***	***p***	
Average Speed†	Pre	1.83	0.116	
	1h	4.50	0.001	**
	2h	6.30	< 0.0005	***
	3h	5.09	0.001	**

	6h	6.36	< 0.0005	***
% of time spent in the bottom of the tank*	Pre	0.49	0.816	
	1h	4.34	0.002	**
	2h	7.56	< 0.0005	***
	3h	6.73	< 0.0005	***
	6h	4.71	0.001	**
% Tank explored†	Pre	1.10	0.376	
	1h	2.76	0.024	*
	2h	4.41	0.002	**
	3h	5.83	< 0.0005	***
	6h	10.08	< 0.0005	***

Table 3: The impact of analgesics on the behaviour of fin clipped female zebrafish. (A) Mixed Model Analysis exploring effects of Treatment (Control, Fin Clip, or four analgesics at various doses), Time (pre-treatment, 1, 2, 3, and 6h after treatment) and their interaction. Post-hoc analysis of (B) the simple effects of Treatment and Time for Average Speed, and analyses of the main effects of (C) Treatment and (D) Time for Percentage time spent at the bottom of the tank and Percentage tank explored are also presented. Analyses include Huynh-Feldt (*) or Greenhouse-Geisser (†) corrections for sphericity as appropriate. Degrees of freedom presented as: term, error.

(A)

**Swim Characteristics**	**Model Term**	**Degrees of Freedom**		***F***	***p***	
Average Speed*	Time × Treatment	39.37,	220.45	1.644	0.014	*
	Time	3.94,	220.45	94.96	< 0.0005	***
	Treatment	10,	56	4.538	< 0.0005	***
% of time spent in the bottom of the tank†	Time × Treatment	23.02,	131.20	0.92	0.570	
	Time	2.30,	131.20	13.15	< 0.0005	***
	Treatment	10,	57	2.142	0.035	*
% Tank Explored*	Time × Treatment	31.95,	182.13	1.155	0.273	
	Time	3.20,	182.13	55.79	< 0.0005	***
	Treatment	10,	57	3.34	0.002	**

(B)

**Treatment**		**Degrees of Freedom**		***F***	***p***	
Control		4.0,	24.0	1.50	0.234	
Fin Clip†		1.5 ,	9.2	24.65	< 0.0005	***
Lidocaine	5mg L^{-1}†	1.5,	8.9	8.54	0.012	*
Flunixin	8mg L^{-1}	4.0,	20.0	4.36	0.011	*

--	4mg L$^{-1\dagger}$	1.2,	6.1	10.80	0.014	*
--	2mg L^{-1}	4.0,	20.0	21.07	< 0.0005	***
Bupivacaine	1mg L^{-1}	4.00,	16.00	27.60	< 0.0005	***
--	0.5mg L$^{-1\dagger}$	1.2,	6.1	9.76	0.017	*
--	0.25mg L^{-1}	4.0,	20.0	12.50	< 0.0005	***
Morphine	48mg L^{-1}	4.00,	16.0	4.87	0.009	**
--	3mg L^{-1}	4.0,	20.0	13.12	< 0.0005	***

**Time**	**Degrees of Freedom**		***F***	***p***	
Pre	10,	68	1.51	0.162	
1h	10,	68	2.50	0.015	*
2h	10,	68	3.39	0.002	**
3h	10,	68	3.90	< 0.0005	***
6h	10,	67	7.19	< 0.0005	***

(C)

**Treatment Comparison**			***p*** **% Bottom Time**	**% Tank Explored**	
**Control**	**Fin Clip**		0.324	0.038	*
	Lidocaine	5mg L^{-1}	1.000	0.999	
	Flunixin	8mg L^{-1}	1.000	1.000	
	--	4mg L^{-1}	0.675	0.933	
	--	2mg L^{-1}	0.957	0.191	
	Bupivicaine	1mg L^{-1}	0.996	1.000	
	--	0.5mg L^{-1}	0.985	1.000	
	--	0.25mg L^{-1}	0.471	0.748	
	Morphine	3mg L^{-1}	1.000	0.683	
	--	48mg L^{-1}	0.999	1.000	
**Fin Clip**	Lidocaine	5mg L^{-1}	0.247	0.002	**
	Flunixin	8mg L^{-1}	0.388	0.161	
	--	4mg L^{-1}	1.000	0.729	
	--	2mg L^{-1}	0.992	1.000	

Bupivicaine	1mg L^{-1}	0.960	0.242
--	0.5mg L^{-1}	0.973	0.079
--	0.25mg L^{-1}	1.000	0.924
Morphine	3mg L^{-1}	0.087	0.952
--	48mg L^{-1}	0.909	0.205

(D)

Time Comparison		***p*** % Bottom Time		% Tank Explored	
Pre	1h	0.003	**	< 0.0005	***
	2h	0.021	*	< 0.0005	***
	3h	< 0.0005	***	< 0.0005	***
	6h	< 0.0005	***	< 0.0005	***
1h	2h	1.000		0.910	
	3h	1.000		0.004	**
	6h	0.773		0.009	**
2h	3h	0.041	*	0.467	
	6h	0.092		0.134	
3h	6h	1.000		1.000	

Table 4. Post hoc testing of Experiment 1 showing only the significant comparisons between treatments (control (C); fin clip (FC), sham, PIT tag, Acid injected subcutaneously into the frontal lips at 1 (AC1), 5 (AC5) and 10% (AC10) before treatment (Pre) and at subsequent time points for the behaviours average swimming speed, percentage (%) time in the bottom and percentage (%) tank explored (NS denotes no significant difference at that time point).

	**Average Speed**		**% Time Bottom**		**% Tank Explored**	
**Time Point**	**Comparisons**	**P**	**Comparisons**	**P**	**Comparisons**	**P**
**Pre**	NS		NS		NS	
**1h**	C & FC	0.017	C & FC	0.040	NS	
	C & AC5	0.003	FC & AC10	0.010		
	C & AC10	0.012	PIT & AC10	0.013		
			AC1 & AC10	0.041		
**2h**	C & FC	0.003	C & FC	0.002	C & FC	0.031
	C & PIT	0.025	C & PIT	0.003	C & AC10	0.029
	C & AC5	0.002	C & AC1	0.039	Sham & FC	0.033
	C & AC10	0.002	FC & AC10	<0.001	Sham & AC10	0.031
	Sham & FC	0.017	PIT & AC10	<0.001		
	Sham & AC5	0.015				
	Sham & AC10	0.012				
**3h**	C & FC	0.002	C & FC	0.015	Sham & FC	0.001
	C & AC5	0.005	C & AC5	0.006	Sham & AC5	0.001
	C & AC10	0.025	Sham & FC	0.033	Sham & AC10	0.002
	Sham & FC	0.018	Sham & AC5	0.013		
	Sham & AC5	0.036	FC & AC10	0.003		
			PIT & AC10	0.028		
			AC1 & AC10	0.044		
				0.001		

			AC5 & AC10			
**6h**	C & FC<br>C & AC5<br>C & AC10<br>Sham & FC<br>Sham & AC5<br>Sham & AC10	0.004<br>0.002<br>0.011<br>0.006<br>0.003<br>0.016	C & FC<br>C & AC5	0.006<br>0.011	C & FC<br>C & AC5<br>C & AC10<br>Sham & FC<br>Sham & PIT<br>Sham & AC5<br>Sham & AC10<br>FC & AC1<br>AC1 & FC5<br>AC1 & AC10	0.004<br>0.001<br>0.001<br><0.001<br>0.019<br><0.001<br><0.001<br>0.040<br>0.009<br>0.012

Table 5. Post hoc testing of Experiment 2 showing only the significant comparisons between treatments (control (C); fin clip (FC) and fin clip fish administered with drugs with pain relieving properties (lidocaine (1mg Lido), bupivacaine at three doses (0.25mg, 0.5mg and 1 mg Bup) flunixin at three doses (2mg, 4mg and 8mg Flu) and morphine at two doses (3mg and 48 mg Mor) before treatment (Pre) and at subsequent time points for the behaviour average swimming speed (NS denotes no significant difference at that time point).

	**Average Speed**	
**Time Point**	**Comparisons**	**P**
**Pre**	NS	
**1h**	NS	
**2h**	C & FC	0.040
	C & 2mg Flu	0.002
**3h**	C & FC	0.005
	C & 2mg Flu	0.015
	FC & Lido	0.003
	2mg Flu & Lido	0.009
**6h**	C & FC	<0.001
	C & 2mg Flu	<0.001
	C & 4mg Flu	0.023
	C & 8mg Flu	<0.001
	C & 0.25mg Bup	0.009
	C & 0.5mg Bup	0.003
	C & 1mg Bup	0.017
	C & 3mg Mor	<0.001

7. Discussion

Behavioural effects of treatment: Female zebrafish were profoundly affected by the invasive procedures employed in the present study with reductions in swimming speed and the amount of the tank explored and an increase in the use of the bottom of the tank. The fin clip and 5/10% acid lip groups had not recovered by the end of the 6 h experiment and their behaviour still differed from pre-treatment behaviour. This prolonged, complicated behavioural change over 6 hours with no recovery indicates that this was not a simple nocifensive reflex[1] but was a substantial modification of the animal's normal behaviour[2]. This response was distinguishable from sham-handled fish whose behaviour did not differ discernibly from controls thus the changes in behaviour are not due to the stress of anaesthesia and handling[1]. These changes were ameliorated by the administration of pain-relieving drugs and thus this information can be used to develop analgesic protocols for fish.

Acetic acid: The injection of increasing concentrations of acetic acid provoked a change in behaviour, similar to that observed in a previous studies[3], which was dose dependent. The injection of 1% acetic acid had only a minor impact on the percentage of tank-explored post 2 and 3h before exploration returned to normal. An increase in concentration up 10%, however, yielded a much greater behavioural response resulting in a significant change across all three behavioural traits. Other studies have used direct observation rather than a behavioural analysis tool but similarly have demonstrated zebrafish reduced their activity after a potentially painful event[3,4]. A reduction in activity and exploration has been observed in zebrafish and trout during painful stimulation[4] and mirror the immobile states and reduced exploration that higher vertebrates can experience in response to a noxious stimulus[5]. Reilly et al.[4] hypothesised that these behaviours may serve to protect the animal from predation when injured by allowing the conservation of energy that may be needed in a fight or flight response and making the individual less conspicuous.

PIT tagging. The insertion of PIT tags resulted in a departure from control behaviour 2 hours after implantation and was characterized by a reduction in average speed and tank exploration with a clear preference for the bottom of the tank. The potential for this procedure to cause pain is considerable given the risk of damage to the musculature of the abdominal cavity, yet the addition of extra weight via the PIT tag could add an energetic cost to movement resulting in the observed reduction in activity. Previous studies investigating the impact of tagging on a broad spectrum of species found no effects on critical swimming velocity[6-8] even when the weight of the tags reached 6-12% of the bodyweight of the individual[8] which are well above the relative weight of the PIT tags (2% of zebrafish bodyweight) used in this study.

Fin clipping. The fin clip procedure resulted in reductions in average speed, tank exploration and a clear preference for the bottom of the tank after treatment. The fin clip procedure resulted in a significant departure from normal behaviour earlier at 1 h and across more time points suggesting that the impact of the fin clip was more immediate and potentially of greater severity relative to the PIT tag. These changes in behaviour have been observed in previous studies in zebrafish[9.10]. Currently, fin clip is a procedure which is deemed mild severity under EU legislation[11] and is believed to result in mild or acute pain for a few hours but our results

show that the responses to fin clipping persists for several hours and as such should be deemed moderately severe. The use of immersion analgesia to alleviate any associated pain would ensure that any pain would be reduced and the procedure was indeed mild.

As with the PIT tag procedure, it is possible that the reduction in activity was related to the physical impediment of having 40% less tail fin as opposed to being a complex response to pain. The complete absence of a tail fin in the no-tail strain of zebrafish resulted in a 65% reduction in critical swimming performance[12], an effect that could account for the observed changes in behaviours linked to activity. If the changes in behaviour are related to the ability to swim with a shorter tail then the administration of analgesics would have no affect; however, certain analgesics at a specific dose helped to ameliorate the behavioural impact of the fin clip thereby confirming the potential for this procedure to be painful.

Impact of drug use: The efficacies of four drugs, from three different classes of analgesics (NSAIDs, opioids and local anaesthetics), differed in their ability to prevent the behavioural change induced by the fin clipping in female zebrafish. Lidocaine (5mg/L) was successful analgesic, since it reduced the effect of the fin clip across all behaviours for the duration of the experiment. Lidocaine treated zebrafish exhibited behaviours that consistently aligned with that observed in the control group even though they had been fin clipped. Studies have found lidocaine to be effective in rainbow trout[13] and zebrafish[10]. Together these findings further validate this drug as a promising analgesic in fish as it has now shown effectiveness in alleviating more than one pain type (chemical and mechanical) in both a cyprinid and a salmonid. The second local analgesic tested was not quite as successful: at 6h fish treated with all doses of bupivacaine displayed behaviour akin to fin clip without analgesia since they were significantly different from controls. In mammalian models the local anaesthetic bupivacaine can have a superior duration of activity[14]; however, this action was not observed here. Instead the highest dose (1 mg/L) did not alleviate the fin clip procedure to the same degree as the lidocaine and can be deemed less effective. Perhaps a higher dose of bupivacaine is required and this should be explored in future studies.

The opioid morphine and NSAID flunixin both proved effective and had a clear dose dependent effect with the highest dose in each case leading to the greatest reduction in fin clip mediated behavioural change. Morphine is effective at ameliorating the impact of noxious stimuli across a large spectrum of vertebrates[15-18] including teleost fish[19]. In this current study, 48mg/L of morphine treated fish behaviour showed average speeds that were similar to control fish. The reverse, however, was observed with the lowest dose of morphine. Although previous studies have largely focused on the injection of morphine, only two have looked at administering morphine via the immersion route in goldfish (*Carassius auratus*)[20,21]. Jansen and Greene[21] demonstrated the rapid uptake of morphine from water in goldfish, however, Newby et al.[20] could not replicate these results and found a much slower rate of uptake. Despite this slow uptake the high dose of morphine (48mg/L) still resulted in a reduction in pain related behaviours; morphine within the water was therefore hypothesised to act centrally[20]. The results from this current study demonstrate the effectiveness of morphine administered via the immersive route, albeit at a high dose, although future work should

examine the uptake kinetics and potential side effects of morphine in zebrafish to help better determine an effective dosage.

The highest dose (8mg/L) of the NSAID flunixin meant fish behaviour did not differ over time and was similar to controls except at the 6h time point. The behaviour average speed in the 8mg/L group were similar to that observed in controls and elevated compared to that observed in the fin clip group. The efficacy seen in the highest dose steadily decreased with dose with the behaviour of the 2mg/L flunixin group resembling that of fin clipped fish and average speed was significantly different from controls at 2, 3 and 6h. NSAID's function through the inhibition of the enzymes arachidonate cyclo-oxygenase 1 (COX-1) and 2 (COX-2); functional genes for both of these enzymes are conserved in zebrafish[22] and levels of the NSAID diclofenac as low as 1 µg/L are known to reduce COX expression in fish[23]. This would suggest that flunixin at 8mg/L is not as effective as lidocaine or morphine or that future studies should test higher doses.

Conclusion. Taken together it would seem from the present study that lidocaine (5mg/L) administered via immersion prior to treatment is the most effective drug to prevent fin clip induced changes. Morphine was also effective but given the cost and regulatory restrictions in its use it may not be widely adopted. We recommend that analgesia is provided for all invasive procedures that cause tissue damage as it is likely they may give rise to the sensation of pain to ensure good welfare. However, where the analgesic drug itself may confound data collection and justifiably cannot be used then experimenters should wait 24 hours after an invasive procedure before beginning behavioural data collection to allow the zebrafish to recover. After this period it has been shown zebrafish behaviour returns to normal after fin clipping[10].

References

1. Rose, J.D. The Neurobehavioral nature of fishes and the question of awareness and pain. *Rev. Fish. Sci*. 10, 1-38 (2002).
2. Sneddon, L.U., Elwood, R.W., Adamo, S.A., and Leach, M.C. Defining and assessing animal pain. *Anim. Behav*. 97, 201-212 (2014).
3. Correia, A.D., Cunha, S.R., Scholze, M., and Stevens, E.D. A Novel Behavioral Fish Model of Nociception for Testing Analgesics. *Pharmaceuticals* 4, 66 (2011).
4. Reilly, S.C., Quinn, J.P., Cossins, A.R., and Sneddon, L.U. Behavioural analysis of a nociceptive event in fish: Comparisons between three species demonstrate. *Appl. Anim. Behav. Sci*. 114, 248-259 (2008).
5. Molony, V., Kent, J.E., and McKendrick, I.J. Validation of a method for assessment of an acute pain in lambs. *Appl. Anim. Behav. Sci*. 76, 215-238 (2002).
6. Ficke, A.D., Myrick, C.A., and Kondratieff, M.C. The effects of PIT tagging on the swimming performance and survival of three nonsalmonid freshwater fishes. *Ecological Engineering* 48, 86-91 (2012).

7. Thorstad, E.B., Rikardsen, A.H., Alp, A., and Okland, F. The Use of Electronic Tags in Fish Research - An Overview of Fish Telemetry Methods. *Turkish Journal of Fisheries and Aquatic Sciences* 13, 881-896 (2013).
8. Brown, R.S., Cooke, S.J., Anderson, W.G., and McKinley, R.S. Evidence to challenge the '2% rule' for biotelemetry. *North American Journal of Fisheries Management* 19, 867-871 (1999).
9. White, L. J., Thomson, J. S., Pounder, K. C., Coleman R. C. and. Sneddon, L. U. The impact of social context on behaviour and the recovery from welfare challenges in zebrafish, Danio rerio. *Animal Behaviour* 132, 189-199 (2017).
10. Schroeder, P. and Sneddon, L. U. Exploring the efficacy of immersion analgesics in zebrafish using an integrative approach. *Applied Animal Behaviour Science* 187, 93-102 (2017).
11. http://ec.europa.eu/environment/chemicals/lab_animals/pdf/report_ewg.pdf
12. Plaut, I. Effects of fin size on swimming performance, swimming behaviour and routine activity of zebrafish *Danio rerio*. *J. Exp. Biol*. 203, 813-820 (2000).
13. Mettam, J.J., Oulton, L.J., McCrohan, C.R., and Sneddon, L.U. The efficacy of three types of analgesic drugs in reducing pain in the rainbow trout, *Oncorhynchus mykiss*. *Appl. Anim. Behav. Sci*. 133, 265-274 (2011).
14. Stock, M.L., and Coetzee, J.F. Clinical Pharmacology of Analgesic Drugs in Cattle. Veterinary Clinics of North America: *Food Animal Practice* 31, 113-138 (2015).
15. Stevenson, G.W., Bilsky, E.J., and Negus, S.S. Targeting pain-suppressed behaviors in preclinical assays of pain and analgesia: Effects of morphine on acetic acid-suppressed feeding in C57BL/6J mice. *Journal of Pain* 7, 408-416 (2006).
16. Ebersberger, A., Anton, F., Tolle, T.R., and Zieglgansberger, W. Morphine, 5-ht2 and 5-ht3 receptor antagonists reduce c-fos expression in the trigeminal nuclear-complex following noxious chemical-stimulation of the rat nasal-mucosa. *Brain Res*. 676, 336-342 (1995).
17. Pert, A., and Yaksh, T. Sites of morphine-induced analgesia in primate brain - relation to pain pathways. *Brain Res*. 80, 135-140 (1974).
18. Lichtenberger, M., and Ko, J. Anesthesia and analgesia for small mammals and birds. *Veterinary Clinics of North America Exotic Animal Practice* 10, 293-315 (2007).
19. Sneddon, L.U. The evidence for pain in fish: the use of morphine as an analgesic. *Appl. Anim. Behav. Sci*. 83, 153-16 (2003).
20. Newby, N.C., Wilkie, M.P., and Stevens, E.D. Morphine uptake, disposition, and analgesic efficacy in the common goldfish (*Carassius auratus*). *Can. J. Zool.-Rev. Can. Zool*. 87, 388-399 (2009).
21. Jansen, G.A., and Green, N.M. Morphine metabolism and morphine tolerance in goldfish. *Anesthesiology* 32, 231-234 (1970).
22. Ishikawa, T., Griffin, K.J.P., Banerjee, U. and Herschman, H.R. The zebrafish genome contains two inducible, functional cyclooxygenase-2 genes. *Biochem Biophys Res Commun*. 352, 181–187 (2007).

23. Mehinto, A.C., Hill, E.M., and Tyler, C.R. Uptake and Biological Effects of Environmentally Relevant Concentrations of the Nonsteroidal Anti-inflammatory Pharmaceutical Diclofenac in Rainbow Trout (Oncorhynchus mykiss). *Environ. Sci. Technol*. 44, 2176-2182 (2010).
